# Biological mechanisms of aging predict age‐related disease co‐occurrence in patients

**DOI:** 10.1111/acel.13524

**Published:** 2022-03-08

**Authors:** Helen C. Fraser, Valerie Kuan, Ronja Johnen, Magdalena Zwierzyna, Aroon D. Hingorani, Andreas Beyer, Linda Partridge

**Affiliations:** ^1^ Department of Genetics, Evolution and Environment Institute of Healthy Ageing University College London London UK; ^2^ Institute of Health Informatics University College London London UK; ^3^ Health Data Research UK London University College London London UK; ^4^ University College London British Heart Foundation Research Accelerator London UK; ^5^ Cologne Excellence Cluster on Cellular Stress Responses in Aging‐Associated Diseases (CECAD) Medical Faculty & Faculty of Mathematics and Natural Sciences University of Cologne Cologne Germany; ^6^ Institute of Cardiovascular Science University College London UK; ^7^ Centre for Molecular Medicine University of Cologne Cologne Germany; ^8^ Max Planck Institute for Biology of Ageing Cologne Germany

**Keywords:** age‐related disease, aging, aging hallmarks, genetics, multimorbidity

## Abstract

Genetic, environmental, and pharmacological interventions into the aging process can confer resistance to multiple age‐related diseases in laboratory animals, including rhesus monkeys. These findings imply that individual mechanisms of aging might contribute to the co‐occurrence of age‐related diseases in humans and could be targeted to prevent these conditions simultaneously. To address this question, we text mined 917,645 literature abstracts followed by manual curation and found strong, non‐random associations between age‐related diseases and aging mechanisms in humans, confirmed by gene set enrichment analysis of GWAS data. Integration of these associations with clinical data from 3.01 million patients showed that age‐related diseases associated with each of five aging mechanisms were more likely than chance to be present together in patients. Genetic evidence revealed that innate and adaptive immunity, the intrinsic apoptotic signaling pathway and activity of the ERK1/2 pathway were associated with multiple aging mechanisms and diverse age‐related diseases. Mechanisms of aging hence contribute both together and individually to age‐related disease co‐occurrence in humans and could potentially be targeted accordingly to prevent multimorbidity.

## Graphical abstract

Using text‐mining, gene set enrichment and network analysis, we found that five aging hallmarks (deregulated nutrient sensing, mitochondrial dysfunction, cellular senescence, stem cell exhaustion and altered intercellular communication) were associated with co‐occurrence of age‐related diseases. Innate and adaptive immunity, Ras‐ERK and intrinsic apoptotic signalling pathways were enriched for all nine aging hallmarks, indicating that multiple diseases may be prevented by targeting these pathways.

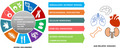

**Abbreviations**
AH: Aging hallmark
ARD: Age‐related disease
EBI: European Bioinformatics Institute
ERK: Extracellular signal regulated kinase
GO: Gene Ontology
GSEA: Gene set enrichment analysis
GWA: Genome‐wide association
IFNg: Interferon gamma
MeSH: Medical Subject Headings
NCBI: National Centre for Biotechnology Information
NHGRI: National Human Genome Research Institute
PMID: PubMed unique Identifier
SNP: Single‐nucleotide Polymorphism

## 1 INTRODUCTION

Age‐associated accumulation of molecular and cellular damage leads to an increased susceptibility to loss of function, disease, and death (Lopez‐Otin et al., 2013). Aging is the major risk factor for many chronic and fatal human diseases, including Alzheimer's disease, multiple cancers, cardiovascular diseases, and type 2 diabetes mellitus (T2DM), which are collectively known as age‐related diseases (ARDs) (Niccoli & Partridge, 2012). However, genetic (Kenyon, 2010), environmental (Austad & Hoffman, 2020), and pharmacological (Partridge et al., 2020) interventions can ameliorate loss of function during aging and confer resistance to multiple age‐related diseases in laboratory animals. Age‐related multimorbidity, the presence of more than one ARD in an individual, is posing a major and increasing challenge to healthcare systems worldwide (Pearson‐Stuttard et al., 2019). An important, open question, therefore, is whether mechanisms of aging can explain ARD co‐occurrence in patients, and hence, whether intervention into these mechanisms could prevent or treat multiple ARDs simultaneously (Franceschi et al., 2018).

**FIGURE 1:**
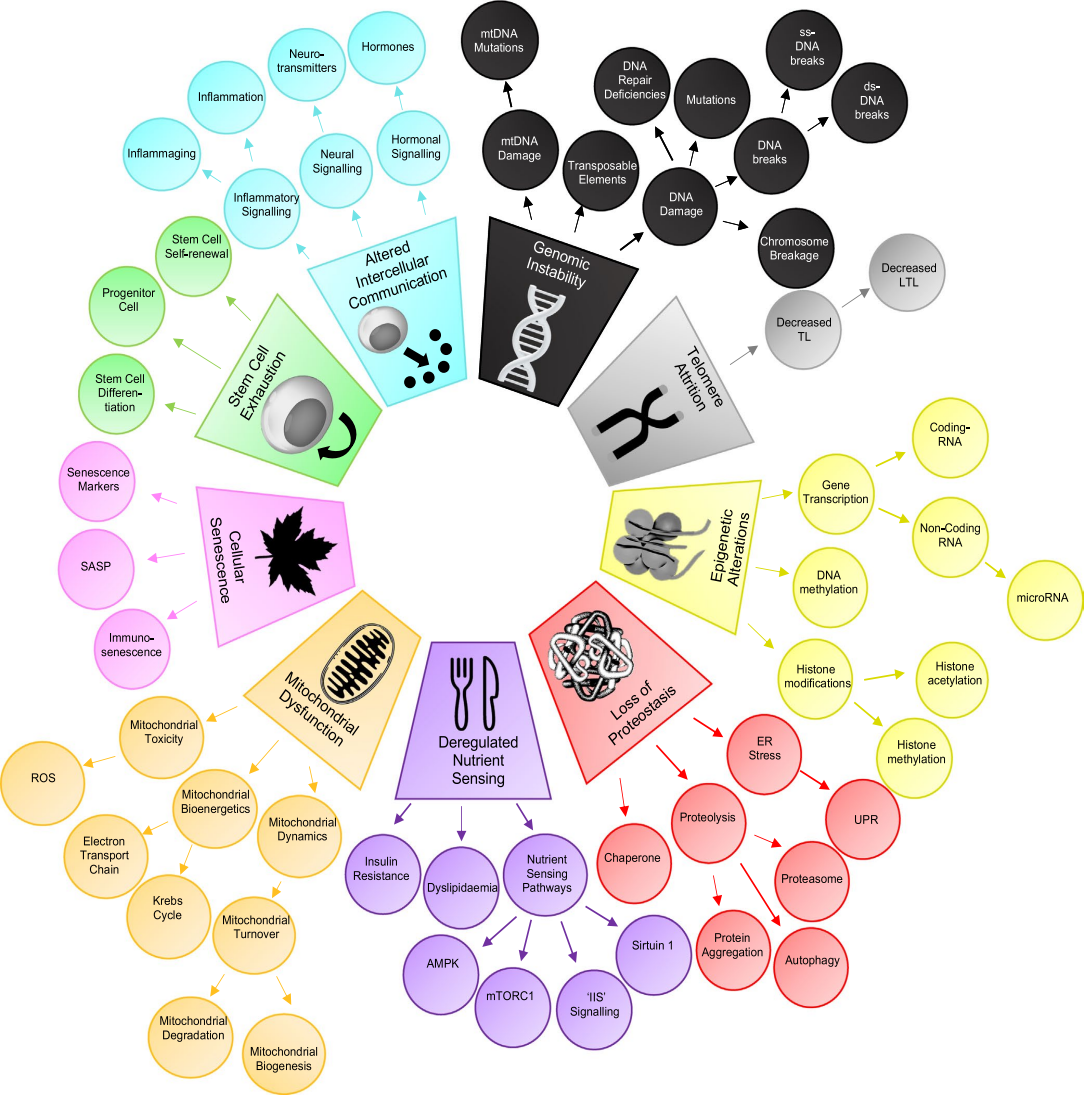
The “Hallmarks of Aging” expanded into a taxonomy. The nine original aging hallmarks were expanded into a taxonomy of 65 related terms and four levels. Figure adapted from Lopez‐Otin et al. (2013). Abbreviations: Table S9

Specific biological mechanisms begin to fail as an individual ages (Lopez‐Otin et al., 2013). Nine major aging processes were summarized as “The Hallmarks of Aging” (Lopez‐Otin et al., 2013): genomic instability, telomere shortening, epigenetic changes, impaired protein homeostasis, impaired mitochondrial function, deregulated nutrient sensing, cellular senescence, exhaustion of stem cells, and altered intercellular communication (Figure 1). Aging hallmarks are not themselves diseases, but they are present in the development and disordered physiology of clinically defined ARDs (Aunan et al., 2016). For example, loss of proteostasis appears to have a prominent role in neurodegenerative disorders, such as Alzheimer's and Parkinson's diseases, which are associated with protein aggregates composed of amyloid‐beta and *α*‐synuclein, respectively (Hou et al., 2019). Genomic instability and epigenetic alterations frequently contribute to development of cancers of, for example, the breast and bowel (Hanahan & Weinberg, 2011). The role of genes in individual human ARDs and ARD multimorbidity has been studied extensively (Amell et al., 2018; Johnson et al., 2015; Zenin et al., 2019), as has the link between individual aging hallmarks and ARDs (Andreassen et al., 2019; Johnson et al., 2015). For example, previous studies have demonstrated that multiple, individual human ARDs share gene ontology (GO) terms linked to aging hallmarks (Johnson et al., 2015). However, whether these underlying mechanisms of aging contribute to ARD co‐occurrence in patients has not previously been investigated. Here, we explore the notion that aging hallmarks may contribute to risk of co‐occurrence of specific ARDs in patients. In model organisms, altering the activity of specific signaling pathways, such as insulin/ insulin‐like growth factor signaling (IIS) (Lopez‐Otin et al., 2013), Ras‐ERK pathway (Slack et al., 2015), immune pathways (Moskalev & Shaposhnikov, 2011), and p53 pathways (Matheu et al., 2007), can delay multiple ARDs and/ or extend lifespan. Therefore, we also explored the notion that common signaling pathways are shared across all aging hallmarks and, thus, may contribute more broadly to multiple ARDs and multimorbidity.

**FIGURE 2:**
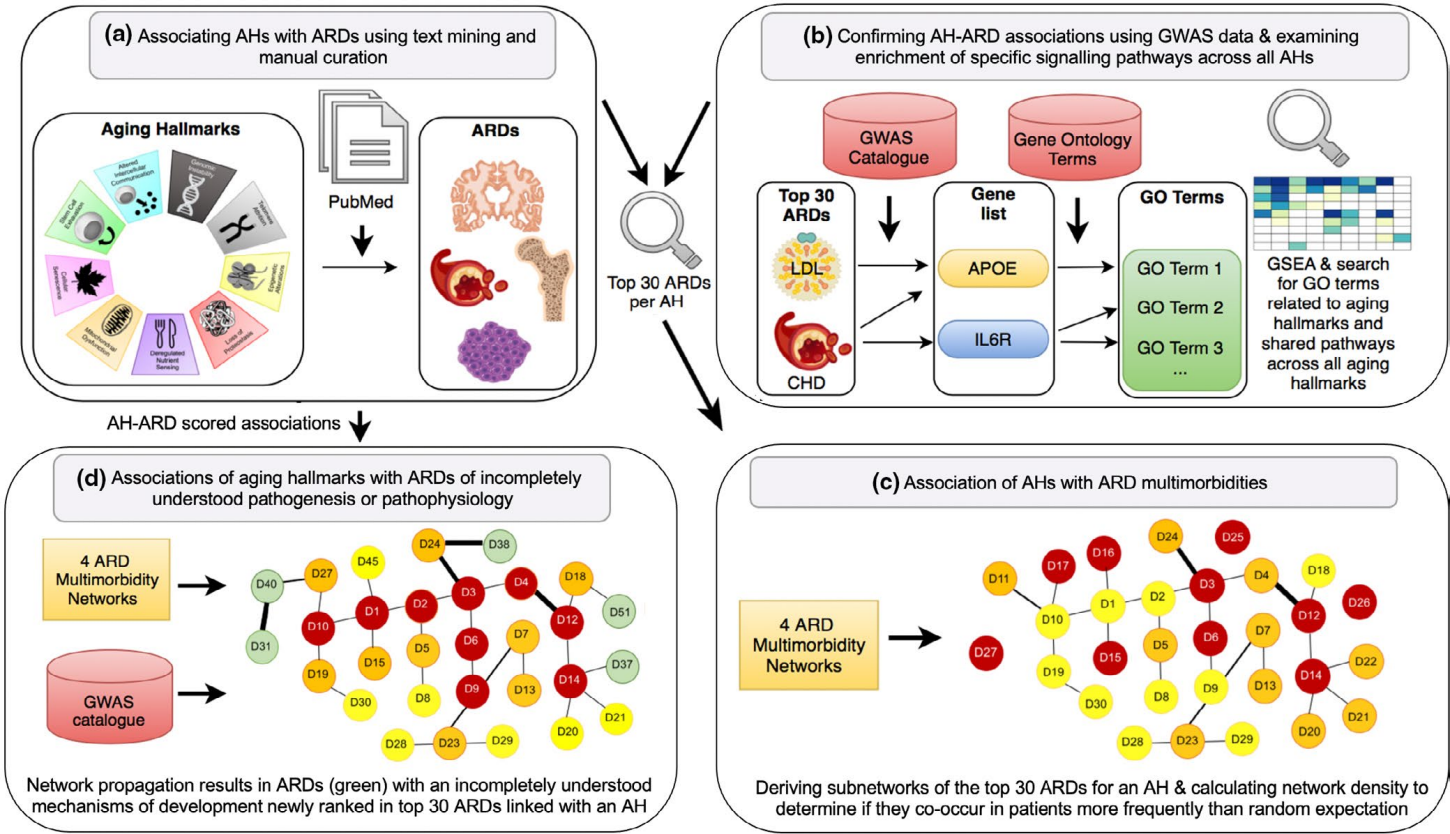
Summary of the methods. (a) Associating aging hallmarks (AHs) with ARDs using text mining. From 1.85 million scientific abstracts, we extracted sentences mentioning and co‐mentioning aging hallmarks and ARDs to derive a score of their association. We kept scores verified using manual curation. The scores were used to identify the top 30 ranked ARDs linked to each aging hallmark. (b) Confirming ARD‐aging hallmark associations using GWAS data and investigating enrichment of specific signaling pathways across all aging hallmarks. We identified the genes linked to each of the top 30 ARDs associated with an aging hallmark from text mining and took the union of genes, which were mapped to encoded proteins forming nine protein lists. We carried out GSEA to identify whether there was significant enrichment of GO terms related to the same aging hallmark as the ARDs were linked to in text mining. We also assessed whether there were significantly enriched signaling pathways across all aging hallmarks. (c) Association of aging hallmarks with ARD multimorbidities. The input data were the top 30 ARDs per aging hallmark from text mining and four ARD multimorbidity networks from age 50 years. We selected subnetworks of the top 30 ARDs per aging hallmark and compared the network density in these subnetworks to random expectation. (d) Associations of aging hallmarks to ARDs with incompletely understood pathogenesis or pathophysiology. We superimposed the aging hallmark‐ARD scored associations from text mining onto the four ARD multimorbidity networks and iterated until convergence. We selected the top 30 ARDs based on the score of the nodes after network propagation and identified significant subnetworks. We identified ARDs with incompletely understood pathogenesis or pathophysiology newly associated with aging hallmarks (green) in the subnetworks and explored genetic data for links to the same aging hallmark

We integrated evidence derived from scientific literature abstracts, genome‐wide association (GWA) studies, and electronic health records to explore the role of aging hallmarks in human ARD co‐occurrence. We began by scoring co‐mentions of aging hallmarks and ARDs in 917,645 scientific literature abstracts and verified the differential aging hallmark‐ARD associations that emerged using manual curation. Using the scores of verified literature aging hallmark‐ARD associations, scaled by the number of mentions of each hallmark and ARD to control for study intensity, we identified the top 30 ranked ARDs specifically associated with each aging hallmark (Figure 2a). To validate these associations independently, we used publicly available GWAS data and found that the annotations of proteins encoded by genes associated with the top 30 ARDs were indeed enriched for processes related to the same aging hallmark (Figure 2b). The resulting associations were then propagated onto previously developed networks of ARD co‐occurrence in clinical data from 3.01 million patients (Kuan, 2020; Kuan et al., 2021). We found that the top 30 ARDs associated with each of 5 of the 9 aging hallmarks co‐occurred more frequently in individual patients than expected by chance (Figure 2c), and these associations were stable over 10‐year age ranges from age 50. Intervention into these individual hallmarks could thus prevent or ameliorate these specific groups of conditions.

In addition to the association of individual aging hallmarks to patterns of ARD co‐occurrence, GO annotation of the GWAS data also indicated that diverse, aging hallmark‐associated ARDs were linked with common signaling pathways. These included innate and adaptive immune, Ras‐ERK, and the intrinsic apoptotic signaling pathways. Interventions into these pathways may therefore have a broad preventative effect for these ARDs.

We also found that aging hallmarks may provide a mechanism for the etiology of ARDs with incompletely understood pathogenesis and/or pathophysiology.

## 2 RESULTS

### 2.1 Associations between aging hallmarks and ARDs in the biomedical literature

Each aging hallmark has a greater role in the development and disordered physiology of certain ARDs and a lesser role in others (Aunan et al., 2016; Lopez‐Otin et al., 2013). If an aging hallmark and ARD are frequently co‐mentioned in the scientific literature, this association could indicate a causal connection between them. We therefore applied text mining to the biomedical literature to identify the ARDs with the highest co‐mentions with each aging hallmark (Figure 2a). As the associations derived from text mining could be confounded by another factor, we verified that the aging hallmark‐ARD associations derived from text mining were direct, using manual curation, and we also sought independent confirmation from GWAS data (Figure 2b).

Our text data consisted of 1.85 million abstracts on human aging extracted from PubMed, termed the “human aging corpus,” and was separated into 20.48 million sentences (Figure 2a). Synonyms of the aging hallmarks and ARDs were needed to maximize identification of relevant sentences in the text data (Pletscher‐Frankild et al., 2015). We therefore developed an aging hallmark taxonomy, so that synonyms and subclasses of an original aging hallmark could be brought into a dictionary for the nine aging hallmarks (Figure 1) (Baker et al., 2017). The starting point for the aging hallmark taxonomy was “The Hallmarks of Aging” (Lopez‐Otin et al., 2013) paper, and the rationale for selection of each taxonomy term is in Table S1. The original nine hallmarks (Lopez‐Otin et al., 2013) were expanded into a taxonomy of 65 related terms and four levels (Figure 1). To develop the ARD dictionary, we used a previous definition, yielding a list of 207 ARDs meeting the criteria (Kuan et al., 2021), from which four ARDs that were not specific enough for scientific literature mining were excluded (Table S2). We then determined if each original aging hallmark synonym and/ or ARD synonym was mentioned in each of the 20.48 million sentences (see Methods, Figure 2a). We excluded 19 ARDs that had fewer than 250 associated sentences in abstracts in the human aging corpus (Table S2). As a co‐occurrence score to quantify aging hallmark‐ARD associations for the remaining 184 ARDs, we used the Ochiai coefficient (Ochiai, 1957), which scores sentences mentioning and co‐mentioning an aging hallmark and an ARD, and adjusts for uneven study density of each aging hallmark and ARD.

**FIGURE 3:**
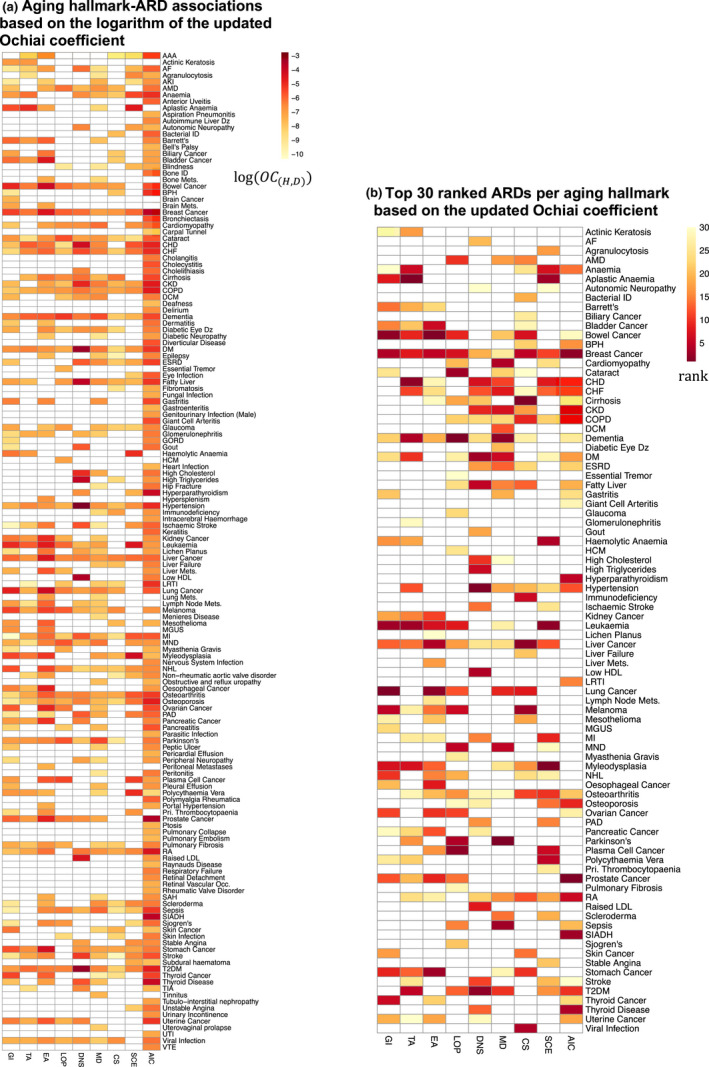
Aging hallmark‐ARD associations from text mining. (a) Aging hallmark‐ARD associations based on the logarithm of the updated Ochiai coefficient. The highest ranked ARDs are in red and lowest ranked in yellow. ARDs with no association are shown in white. (b) The top 30 ranked ARDs for each aging hallmark. 1st (dark red) to 30th (light yellow) ranked ARDs for a given aging hallmark are highlighted. ARDs not ranked in the top 30 are shown in white. Abbreviations: Table S9

Age‐related diseases and aging hallmarks with higher Ochiai coefficients are likely to be related in some way, but the type of relationship, for instance a causal connection, is not known (Jensen et al., 2006). Therefore, we manually examined sentences co‐mentioning each aging hallmark‐ARD pair to determine the type of relationship (Yang et al., 2016). We manually examined co‐mentioning sentences until we had encountered a sufficient number (see Methods) that correctly reported that an aging hallmark had a role in the development or disordered physiology of a disease (Table S4). Aging hallmark‐ARD combinations with insufficient evidence of association from manual curation were set to zero and the Ochiai coefficient associating each aging hallmark and ARD was updated. The updated Ochiai coefficients were then sorted in descending order to provide a rank for association of each ARD with each aging hallmark (Figure 3a). We selected the top 30 ARDs associated with each aging hallmark (Figures 2a and 3b) as a prioritized and sufficiently large number to explore in multimorbidity networks.

The Ochiai coefficients showed clear patterns of association between specific aging hallmarks and ARDs (Figure 3a,b). For instance, disorders frequently mentioned in association with genomic instability and epigenetic alterations were primary malignancies, such as lung cancer, bowel cancer, and leukemia (Figure 3b). This was as expected, since “genomic instability and mutation” are hallmarks of cancer and epigenetic alterations are important in cancer development and progression (Hanahan & Weinberg, 2011; Kanwal & Gupta, 2012). Highly ranked ARDs for telomere attrition and stem cell exhaustion were hematological disorders, including aplastic anemia, anemia, and myelodysplasia (Figure 3b) (Lopez‐Otin et al., 2013). There were strong associations between proteostasis and neurodegenerative disorders including dementia, Parkinson's disease, and motor neurone disease (MND), which are indeed associated with amyloid‐beta aggregates, *α*‐synuclein aggregates, and dipeptide‐repeat polymers, respectively (Figure 3b) (Hou et al., 2019; Vanneste et al., 2019). Mitochondrial dysfunction was strongly associated with neurodegenerative disorders and cardiomyopathy, again showing that our approach could recapture established associations (Figure 3b) (Aunan et al., 2016; Johri & Beal, 2012). Highly ranked ARDs for cellular senescence included immunodeficiency, which is associated with immunosenescence, and cancers, which are exacerbated by the senescence‐associated secretory phenotype (Figure 3b) (Gonzalez‐Meljem et al., 2018; McLachlan et al., 1995). Highly ranked disorders for deregulated nutrient sensing were high triglycerides, low high‐density lipoprotein (HDL) cholesterol, hypertension, and type 2 diabetes mellitus (T2DM) (Figure 3b). These ARDs comprise the metabolic syndrome, which is strongly associated with insulin resistance (Lann & LeRoith,). Altered intercellular communication was associated with specific malignancies and autoimmune disorders, such as prostate cancer and rheumatoid arthritis (RA), respectively (Figure 3b) (Kryvenko et al., 2012). Thus, our text mining approach correctly captured many molecular and cellular processes known to be involved in the respective ARD etiology and, importantly, confirmed that aging hallmark‐ARD associations were highly non‐random.

### 2.2 Confirmation of ARD‐aging hallmark associations from GWAS data

We next used genetic information to obtain independent confirmation of the aging hallmark‐ARD associations derived from text mining. We assessed whether proteins encoded by genes associated with top 30 ARDs showed significant enrichment of GO terms related to the same aging hallmark on GSEA (Figure 2b). We linked the top 30 ARDs per aging hallmark to genes using the GWAS catalog (Buniello et al., 2019) (Figure 2b), thus obtaining 9 gene lists (Figure 2b). As GO terms are mapped to gene products, we mapped each of the protein‐coding genes to a single protein typically representing the canonical isoform, resulting in nine “protein lists” (Table 1a) (Szklarczyk et al., 2019). We then carried out GSEA to test for significant enrichment of biological process GO terms related to the same aging hallmark (Figure 2b, Figure S1a‐i). The GWAS catalog is associated with PMIDs, and we avoided any risk of circularity by removing the PMIDs that intersected between studies included from the GWAS catalog and the 917,645 scientific titles/ abstracts mentioning aging hallmarks and/or ARDs. Thus, this approach to verifying aging hallmark‐ARD associations was independent of the literature‐based method.

**TABLE 1:** Number of proteins in each aging hallmark protein list and number of proteins in each list linked to the five significant signaling pathways

Aging hallmark	a. Total number of proteins in protein list	Number of proteins in protein list linked to signaling pathway(expected number)				
		b. IFN‐γ	c. T‐cell	d. T‐cell (positive regulation)	e. ERK1/2 (positive regulation)	f. intrinsic apoptotic
GI	511	9 (2.7)***	13 (3.7)***	3 (0.4)**	15 (6.0)**	7 (1.4)***
TA	872	19 (4.7)****	21 (6.3)***	5 (0.7)***	27 (10.3)****	8 (2.4)***
EA	658	14 (3.5)****	20 (4.7)****	4 (0.5)**	17 (7.8)**	7 (1.8)***
LOP	817	16 (4.4)****	17 (5.9)***	4 (0.6)**	26 (9.7)****	6 (2.2)*
DNS	1212	20 (6.5)**	26 (8.7)****	4 (1.0)*	31 (14.3)****	7 (3.3)*
MD	1058	20 (5.7)****	24 (7.6)***	5 (0.8)***	31 (12.5)****	8 (2.9)**
CS	594	10 (3.2)**	17 (4.3)***	3 (0.5)**	16 (7.0)**	9 (1.6)****
SCE	680	17 (3.7)****	17 (4.9)**	4 (0.5)**	23 (8.0)****	7 (1.8)***
AIC	809	14 (4.3)***	19 (5.8)***	3 (0.6)*	24 (9.6)****	7 (2.2)**
Total (union of encoded proteins)		25	30	5	40	9
Total (union of mapped ARDs)		21	19	9	22	11

Note

We identified the genes linked to each of the top 30 ARDs associated with an aging hallmark from text mining. We took the union of genes leading to nine gene lists. Protein‐coding genes within each gene list were mapped to proteins forming nine protein lists. (a) Total number of proteins in each protein list. The associated aging hallmark from text mining represents the rows in the “aging hallmark” column (i.e., genomic instability (GI), telomere attrition (TA), epigenetic alterations (EA), loss of proteostasis (LOP), cellular senescence (CS), deregulated nutrient sensing (DNS), mitochondrial dysfunction (MD), stem cell exhaustion (SCE), and altered intercellular communication (AIC)). We next carried out GSEA followed by a search for GO terms mentioning “pathway” or “cascade,” which showed significant enrichment of five pathways across all aging hallmark protein lists represented in (b‐f). The number of proteins in each protein list linked to the GO terms: (b) “IFN‐γ‐mediated signaling pathway,” (c) “T‐cell receptor signaling pathway,” (d) “positive regulation of T‐cell receptor signaling pathway,” (e) “positive regulation of the ERK1/2 cascade,” and (f) “intrinsic apoptotic signaling pathways in response to DNA damage by p53 class mediator,” compared to the expected number (*p < 0.05, ** *p* < 0.01, ***p < 0.001, ****p < 0.0001). The “total” rows show the union of proteins from all nine protein lists and the union of mapped ARDs.

We next tested whether biological processes related to each aging hallmark were indeed significantly enriched in the protein list representing the top 30 ARDs associated with that hallmark (Figure 2b, Figure S1a‐i). Both 511 and 1212 proteins were associated with each of the aging hallmarks (Table 1a). We carried out GSEA and searched for GO terms related to each aging hallmark (Figure S1a‐i). We identified significant enrichment of terms related to the same aging hallmark as was associated with the ARDs via text mining (Figure S1a‐i). For example, “DNA damage response,” “telomere maintenance,” “regulation of autophagy,” “replicative senescence,” “glucose homeostasis,” “regulation of mitochondrion fission,” and “stem cell differentiation” were significantly enriched in the genomic instability, telomere attrition, loss of proteostasis, cellular senescence, deregulated nutrient sensing, mitochondrial dysfunction, and stem cell exhaustion protein lists, respectively (Figure S1a,b,d‐h). The altered intercellular communication protein list showed significant enrichment of processes related to hormone synthesis and inflammatory response while the epigenetic alteration protein list showed significant enrichment of terms related to histone acetylation (Figure S1c, i). Thus, the protein lists derived from the aging hallmark‐associated gene lists were significantly enriched for annotations related to their own aging hallmark. Therefore, analysis of GWAS data confirmed the specific associations between aging hallmarks and ARDs that had been found from the literature co‐occurrence scores (Figure 2a,b).

### 2.3 Enrichment of signaling pathways across all aging hallmarks

**FIGURE 4:**
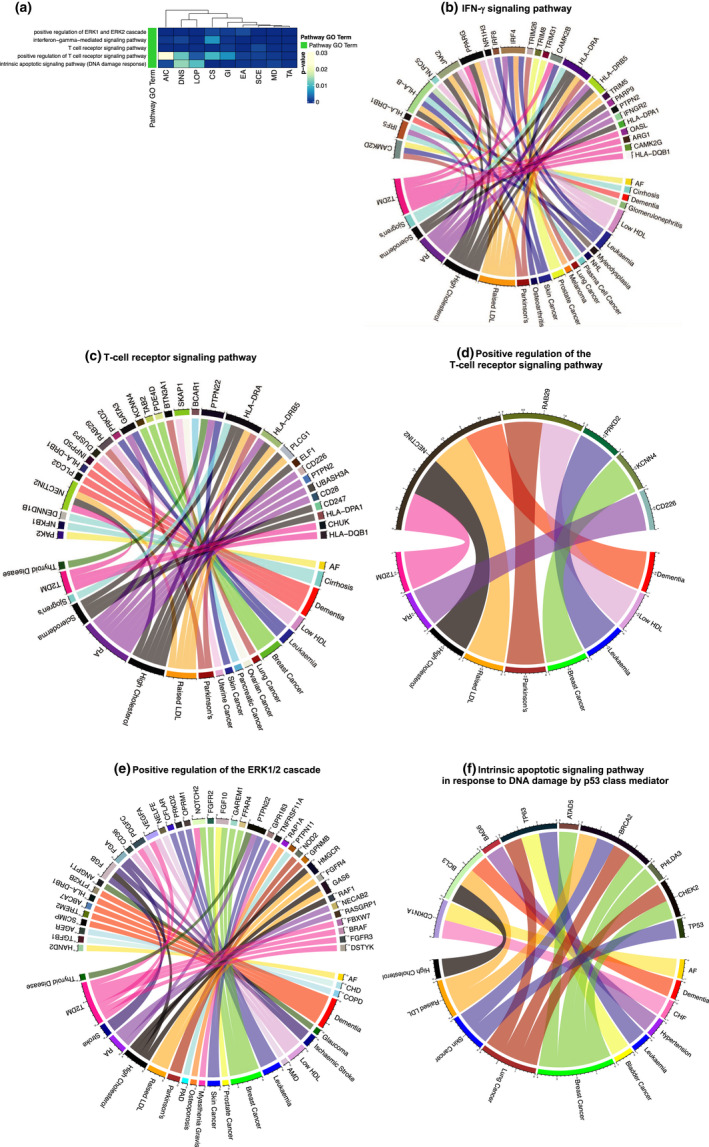
Significantly enriched signaling pathways across all aging hallmark protein lists. (a) *p*‐values of enriched signaling pathways across all aging hallmarks. We identified the genes linked to each of the top 30 ARDs associated with an aging hallmark from text mining and took the union of genes. These were mapped to encoded proteins forming nine protein lists. The associated aging hallmark from text mining represents the column labels of the heatmap. We carried out GSEA and searched for GO terms related to signaling pathways. Five signaling pathways were significantly enriched across all aging hallmark protein lists. (b‐f) The union of proteins/ genes associated with each of the five significantly enriched pathways was derived and they were linked to their associated ARDs. These are shown in the circos plots representing: (b) IFN‐γ‐mediated signaling pathway, (c) T‐cell receptor signaling pathway, (d) positive regulation of T‐cell receptor signaling pathway, (e) positive regulation of the ERK1/2 cascade, and (f) the intrinsic apoptotic signaling pathway in response to DNA damage by p53 class mediator. Abbreviations: Table S9

Our literature mining revealed highly specific associations between ARDs and aging hallmarks, and these were independently confirmed by GWAS data. However, hallmarks of aging are part of a complex nexus of failure of molecular and cellular processes, are not independent of each other, and may share some common underlying signaling pathways. Therefore, we explored whether common signaling pathways were shared across all aging hallmark protein lists and, thus, contribute to the development of multiple aging hallmark‐associated ARDs. For the ARDs that were associated with specific hallmarks and that were present in our GWAS analysis, there was clear evidence from the GWAS data for commonalities in the signaling cascades and pathways across all aging hallmark protein lists (Figure 4a). GSEA followed by search for GO terms mentioning “pathway” or “cascade” showed that five pathways were significantly enriched in all aging hallmark protein lists (Figure 4a, Table 1b‐f). Three were linked to the innate and adaptive immune system, including the “interferon‐γ‐mediated signaling pathway” and the “T‐cell receptor signaling pathway” and to its “positive regulation” (Figure 4a, Table 1b‐d). These pathways are interconnected, as interferon‐γ is a cytokine produced by multiple immune cells including cells of the adaptive immune system, such as T cells (Yen et al., 2000). “Positive regulation of the ERK1/2 cascade” and the “intrinsic apoptotic signaling pathway in response to DNA damage by a p53 class mediator” were also significantly enriched across all aging hallmark protein lists (Figure 4a, Table 1e, f).

To explore these common pathways further, we derived the union of proteins associated with each of the GO terms across all aging hallmarks, mapped them to their underlying genes, and linked them to their associated ARDs (Table 1b‐f). A total of 21 ARDs were linked to 25 genes encoding proteins associated with the interferon‐γ pathway (Figure 4b, Table 1b), 19 to 30 genes encoding proteins associated with the T‐cell receptor signaling pathway (Figure 4c, Table 1c), 9 to 5 genes encoding proteins associated with positive regulation of the T‐cell receptor signaling pathway (Figure 4d, Table 1d), 22 to 40 genes encoding proteins associated with the ERK1/2 cascade (Figure 4e, Table 1e) and 11 to 9 genes encoding proteins associated with the intrinsic apoptotic signaling pathway (Figure 4f, Table 1f). These signaling cascades are therefore implicated in the etiology of these diverse, aging‐hallmark‐associated ARDs.

### 2.4 Association of aging hallmarks with ARD multimorbidities

We next explored the possible role of aging hallmarks in the co‐occurrence of two ARDs in the same patient, known as multimorbidity (Figure 2c). To do this, we assessed whether ARDs associated with the same aging hallmark occurred more frequently in the same patient than random pairs of ARDs. We used previously created multimorbidity networks (Kuan, 2020) reflecting non‐random co‐occurrence of two diseases in the same patient. The multimorbidity networks were created for different age classes by binning electronic health records of 3.01 million individuals into nine 10‐year age intervals (Kuan, 2020; Kuan et al., 2019, 2021). Within each age interval, significantly co‐occurring disease pairs were linked in the respective network (see Methods) (Kuan, 2020). The stratification by age accounts for the fact that occurrence (Kuan et al., 2019) and co‐occurrence (Kuan, 2020) of diseases change with age. Since we were particularly interested in ARDs, we used the four networks for the age groups of 50 years and over for subsequent analyses because 170 of the 184 ARDs had a median age of onset ≥50 years (Figure 2c) (Kuan et al., 2021). Thereby, we obtained four networks of 184 ARDs (Table S6) (Kuan, 2020; Kuan et al., 2021).

We next assessed whether the ARDs associated with each aging hallmark were more likely to co‐occur as multimorbidities in patients than expected by chance. We selected the top 30 ARDs for each aging hallmark and extracted the subnetworks consisting of those 30 diseases (Figures 2c and 3b), resulting in 36 subnetworks for the four age‐specific ARD multimorbidity networks and the nine aging hallmarks. A higher observed network density than expected by chance indicates that there are more edges than expected, and hence that the ARDs within the subnetwork are more frequently multimorbidities than random ARD sets of the same size. In order to estimate the statistical significance of such differences, we performed 20,000 random permutations of the network topology and obtained a background distribution of network densities. Next, we compared the network densities of the top 30 ARDs for each hallmark with that background distribution to obtain *p*‐values (see Table 2 for details).

**TABLE 2:** Network density of subnetworks of the top 30 ranking ARD nodes compared to random expectation for age categories 50–59 years, 60–69 years, 70–79 years, and ≥80 years

Aging hallmark	ARD network density			
	50–59 years	60–69 years	70–79 years	≥80 years
Genomic instability	0.0805	0.0989	0.0897	0.0782
Telomere attrition	0.1126	0.1218	0.1103	0.1011
Epigenetic alterations	0.0851	0.0759	0.0782	0.0713
Loss of proteostasis	0.0897	0.0805	0.0828	0.0552
Deregulated nutrient sensing	0.2598****	0.2644****	0.2368****	0.2207****
Mitochondrial dysfunction	0.1655*	0.1471*	0.1356*	0.1080*
Cellular senescence	0.1379*	0.1494*	0.1195*	0.0989*
Stem cell exhaustion	0.2092***	0.2000***	0.1724***	0.1609****
Altered intercellular comm.	0.2000***	0.1839**	0.1540**	0.1333**

Note

The number of times the network density from permutations (*n* = 20,000) was greater than or equal to the true network density for that aging hallmark was used to calculate the *p*‐value. The *p*‐value was corrected for multiple testing across the 4 age categories per aging hallmark using the Benjamini–Hochberg procedure (*p < 0.05, ** *p* < 0.01, ***p < 0.001, ****p < 0.0001).

**FIGURE 5:**
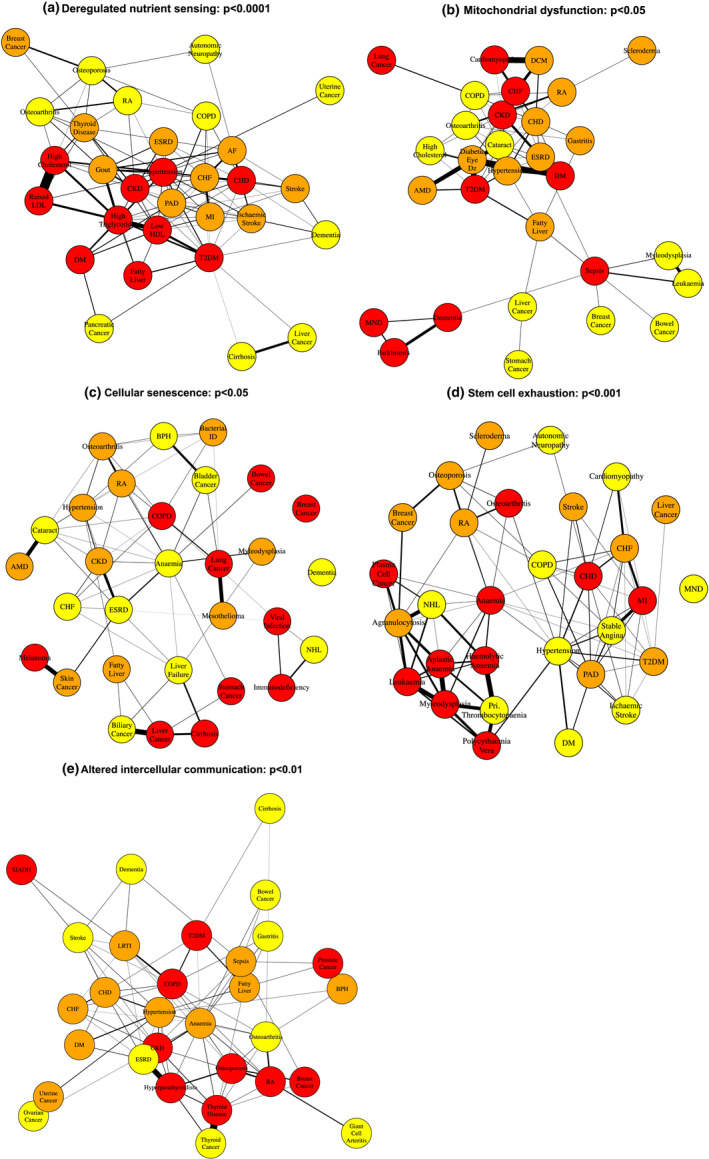
Subnetworks containing nodes representing the top 30 ranked ARDs for each aging hallmark (50–59 year age category). The (a) deregulated nutrient sensing, (b) mitochondrial dysfunction, (c) cellular senescence, (d) stem cell exhaustion, and (e) altered intercellular communication subnetworks. Nodes are colored by ARD ranking for a given aging hallmark: the 1st to 10th ranked in red, the 11th to 20th ranked in orange, and the 21st to 30th ranked in yellow. Abbreviations: Table S9

For five of nine aging hallmarks, namely deregulated nutrient sensing (*p* < 0.0001), mitochondrial dysfunction (*p* < 0.05), cellular senescence (*p* < 0.05), stem cell exhaustion (*p* < 0.001), and altered intercellular communication (*p* < 0.01), the nodes representing the top 30 associated ARDs were connected by more edges than expected by chance across all age categories (Table 2, Figure 2c). The ARDs associated with these five aging hallmarks thus co‐occurred in individual patients more frequently than expected by chance and these associations were stable over 10‐year age ranges from age 50 years (Figure 5a‐e, Table 2). For example, the deregulated nutrient sensing multimorbidity subnetwork contained nodes connected by edges representing the progression of known multimorbidities, such as type 2 diabetes mellitus with fatty liver (Figure 5a) (Kneeman et al., 2012). These non‐random associations suggest that these five aging hallmarks do indeed have a role in the development of ARD multimorbidity in patients (Table 2).

### 2.5 Associations of aging hallmarks with ARDs with incompletely understood pathogenesis or pathophysiology

The analysis above suggests that ARDs that are tightly connected in the multimorbidity networks are more likely affected by the same hallmark than random pairs of diseases. Thus, we speculated that this association could be used to identify hallmark‐ARD associations that were so far unknown, that is, based on the fact that many neighboring ARDs in the network are associated with a common hallmark (“guilt by association”) (Cowen et al., 2017). Therefore, we focused on ARDs with incompletely understood pathogenesis or pathophysiology, that were not originally ranked in the top 30 ARDs associated with a hallmark, but where the hallmark may nonetheless contribute to etiology.

For each aging hallmark, we superimposed the aging hallmark‐ARD co‐occurrence scores (or updated Ochiai coefficients) from text mining onto the respective ARD nodes in each of the four multimorbidity networks (Figure 2d). The scores were then smoothed over the network, which amplifies regions where ARDs have higher co‐occurrence scores with a given aging hallmark and dampens regions with lower scores (Cowen et al., 2017) and thus assigns relatively high scores to ARDs that are surrounded in the network by ARDs associated with a common hallmark. Since this process changes the ARD‐hallmark associations of all diseases in the network, it also changes the ranking of ARDs associated with each aging hallmark (Figure 2d). We identified those subnetworks with a significantly greater network density than random expectation and identified newly prioritized ARDs within them (Table S7).

Two ARDs with incompletely understood mechanism of pathogenesis or pathophysiology were newly ranked among the top 30 ARDs, namely essential tremor and Bell's Palsy (Table S7, Figure S2a,b) (Louis, 2014; Zhang et al., 2020). Essential tremor is a neurological disorder characterized by an involuntary, rhythmic tremor and was newly prioritized as a top 30 ARD associated with mitochondrial dysfunction (Figure S2a). It has previously been associated with mitochondrial abnormalities; however, the degree of their role is unclear (Unal Gulsuner et al., 2014). This disorder also has genetic evidence of association with five genes (i.e., STK32B, NAT2, LINGO1, CTNNA3, and LRRTM3) at genome‐wide significance. However, we cannot exclude that the association is a consequence of initial misdiagnosis, such as of Parkinson's disease as essential tremor (Thenganatt & Louis, 2012). Bell's palsy was newly prioritized as a top 30 ARD associated with deregulated nutrient sensing, which has previously been reported to be associated with prognosis of the Bell's palsy (Karagoz et al., 2020). However, the association may also be a consequence of initial misdiagnosis of diabetic mononeuropathy as Bell's palsy (Figure S2b) (Pecket & Schattner, 1982). There were no reported genetic associations with Bell's palsy in the GWAS catalog at genome‐wide significance. Overall, our findings indicate that aging hallmarks may contribute to a better understanding of disease etiology.

## 3 DISCUSSION

The contribution of aging hallmarks to co‐occurrence of ARDs in humans is largely unexplored. We have addressed the issue by combining aging hallmark‐ARD associations derived from text mining, independently verified using genetic data, with disease networks derived from electronic health records.

First, we explored patterns of association between specific aging hallmarks and ARDs. We text mined 917,645 literature abstracts, followed by manual curation, and found strong, non‐random associations between ARDs and aging hallmarks.

By integrating our findings with networks of ARD co‐occurrence in patients, we found that five aging hallmarks were indeed non‐randomly associated with specific ARD co‐occurrence networks. Deregulated nutrient sensing, mitochondrial dysfunction, cellular senescence, stem cell exhaustion, and altered intercellular communication were associated with the co‐occurrence of ARDs in individual patients more than expected by chance. Reassuringly, these aging hallmarks were associated with ARD multimorbidity across all four decadal age ranges, and the associations were often highly significant. Overall, these findings indicate that therapies targeted at each of these five aging hallmarks may prove to be beneficial in the prevention of their associated ARD multimorbidities in humans. For instance, sirolimus and related compounds inhibit the TORC1 complex in the nutrient‐sensing network (Castillo‐Quan et al., 2019) and can both extend healthy lifespan in model organisms (Correia‐Melo et al., 2019) and boost the response to vaccination against influenza in elderly people (Mannick et al.,). Senolytics and senescence‐associated secretory phenotype (SASP) modulators eliminate senescent cells and inhibit the SASP, respectively, and thus target the cellular senescence hallmark (Gonzalez‐Meljem et al., 2018), and can both improve tissue health during aging and increase lifespan in mice (Xu et al., 2018) and may prevent cellular senescence‐associated ARD multimorbidities (Khosla et al., 2020). It will be important in any clinical trials that target these aging mechanisms pharmacologically to consider potential effects on the multiple associated ARDs.

In model organisms, targeting common signaling pathways delays the onset of ARDs and extends lifespan (Lopez‐Otin et al., 2013; Matheu et al., 2007; Moskalev & Shaposhnikov, 2011; Slack et al., 2015). Specific signaling pathways are intertwined with the aging hallmarks, for example, the IIS pathway is associated with the deregulated nutrient sensing aging hallmark (Lopez‐Otin et al., 2013). Aging hallmarks are not independent of each other with, for instance, DNA damage and telomere shortening contributing to cellular senescence (Fyhrquist et al., 2013) and loss of stem cell function (Behrens et al., 2014). Thus, different aging hallmarks may share some common underlying pathways, which will hence contribute to the development of multiple, aging‐hallmark‐associated ARDs. Five signaling pathways/ cascades were significantly enriched across the protein lists for all nine aging hallmarks. These pathways are therefore likely to play a key role in the etiology of ARDs. Among these five signaling pathways, three were involved in the innate and/ or adaptive immune response. The underlying genes were derived from ARDs comprising metabolic syndrome disorders, autoimmune disorders, and cancers, thus highlighting the importance of the immune response across multiple ARDs (Johnson et al., 2015). The “intrinsic apoptotic signaling pathway in response to DNA damage by a p53 class mediator” was also significantly enriched across all aging hallmark protein lists. The underlying genes were derived from multiple cancers and metabolic syndrome disorders (Hanahan & Weinberg, 2011; Mercer et al., 2010). The ERK1/2 pathway regulates many processes including cell survival, metabolism, and inflammation (Sun & Nan, 2017) and was significantly enriched across all aging hallmark protein lists. The underlying genes were derived from 22 aging hallmark‐associated ARDs (Figure 4e), and indeed, activation of the ERK1/2 pathway has been suggested to play a role in these ARDs either directly or through their risk factors. For example, increased activity of the ERK1/2 pathway has been identified in type 2 diabetes mellitus (Tanti & Jager, 2009) and hypertension (Roberts, 2012), which are major risk factors for cardiovascular disorders. Additionally, activating mutations upstream of ERK1/2 contribute to over fifty percent of human cancers (Montagut & Settleman, 2009). Increased phosphorylation of cellular ERKs has also been identified in the thyroid disorder, hypothyroidism (Suarez et al., 2010), and in atrial fibrillation (Goette et al., 2000). Furthermore, ERK1/2 inhibition reduces beta‐amyloid neurotoxicity in Alzheimer's disease (Sun & Nan, 2017), decreases inflammation and apoptosis in stroke patients (Sun & Nan, 2017), and prevents rheumatoid arthritis in mouse models (Ohori, 2008). Interestingly, the ERK1/2 cascade is linked to aging in model organisms and the MEK inhibitor, Trametinib, prolongs lifespan in *Drosophila* (Slack et al., 2015). Thus, our analysis suggests that inhibition of the ERK1/2 pathway could prevent up to 22 human aging hallmark‐associated ARDs.

Using network propagation, we identified ARDs with incompletely understood pathogenesis where aging hallmarks may contribute to their development. Essential tremor has previously been associated with mitochondrial abnormalities, but the degree of their role is unclear (Louis, 2014; Unal Gulsuner et al., 2014). We found that essential tremor co‐occurred with many ARDs strongly linked to mitochondrial dysfunction implying this is in fact a key pathogenic mechanism in essential tremor. However, we cannot exclude the association as a consequence of initial misdiagnosis, such as of Parkinson's disease as essential tremor (Thenganatt & Louis, 2012). Our findings were also supported by genetic data, as essential tremor is also associated with the variant N‐acetyltransferase 2 (NAT2) gene. NAT2 is associated with insulin resistance (Knowles et al., 2015), and deficiency of the mouse orthologue (i.e., NAT1) has also been associated with mitochondrial dysfunction (Chennamsetty et al., 2016). Therefore, aging hallmarks may contribute to the development of ARDs with incompletely understood mechanisms of development.

A potential limitation is that, because certain ARDs occupy more of the scientific research effort, there is a risk that they would be more frequently included in the top 30 ARDs associated with aging hallmark and, therefore, included in multimorbidity subnetworks. To reduce the risk of this, we adjusted for uneven study density on each ARD by using a co‐occurrence score based on the Ochiai co‐efficient. A further potential limitation of the literature search is that it may have missed some associations between aging hallmarks and ARDs because they have been little studied. However, similar associations emerged from GSEA using GO annotations of proteins encoded by genes linked to the top 30 ARDs. We were thus able to detect signals that allow us to conclude that: (1) individual hallmarks contribute to multiple diseases, (2) highlight which hallmarks and pathways contribute to which diseases and (3) direct future research toward interventions on the hallmarks (and associated pathways) to tackle the prevention/management of these multiple diseases. An additional potential limitation is that ARD multimorbidities may be connected in electronic health records due to incorrect initial diagnosis, which may complicate the evaluation of incompletely explained ARDs. These limitations will be overcome as our knowledge of the aging hallmarks, ARD multimorbidities, and genes underlying ARDs improves.

Our study provides evidence for the role of aging hallmarks in the etiology of human ARD multimorbidities and ARDs with incompletely understood pathogenesis. We also raise the possibility that multiple ARDs may be prevented by targeting common signaling pathways, such as the innate and adaptive immune pathways, the intrinsic apoptotic signaling pathway, and the ERK1/2 pathway. Future work will determine whether a prophylactic agent or cure for human ARD multimorbidities can be developed by targeting each of five aging hallmarks.

## 4 METHODS

The methods are summarized in Figure 2.

### 4.1 Information retrieval of the “human aging corpus”

A set of primary research articles (or corpus) on human aging was required for text mining. Our corpus was developed by defining inclusion and exclusion criteria followed by retrieving 1.93 million PubMed identifiers (PMIDs) of abstracts meeting those criteria from PubMed (Table S8a,b). The 1.93 PMIDs representing title/ abstracts on human aging meeting our search criteria were retrieved from the PubMed database using the Biopython Entrez application programming interface (Cock et al., 2009) on April 10, 2020. Next, the 2019 PubMed baseline contains over 29 million abstracts and was downloaded in Extensible Mark‐up Language (XML) format. Data were extracted from the XML files to produce separate, comma‐separated values (CSV) files containing 29,138,919 million rows and six columns including titles, abstracts, and PMIDs. The rows containing the 1.93 million PMIDs of the human aging corpus were identified. PMIDs associated with missing data were eliminated, and, subsequently, the text data were cleaned. This gave 1.85 million abstracts representing the “human aging corpus,” which were tokenized into 20.48 million sentences.

### 4.2 Information extraction by dictionary‐based methods with co‐occurrence scoring

#### 4.2.1 Aging hallmark dictionary

An aging hallmark taxonomy was developed to maximize retrieval of relevant literature on each aging hallmark from PubMed (Figure 1). We modeled our methodology on the approach used previously to develop a cancer hallmarks taxonomy (Baker et al., 2017; Hanahan & Weinberg, 2011). The starting point for the taxonomy was the original “The Hallmarks of Aging” (Lopez‐Otin et al., 2013) paper from which we selected relevant subcategories of the nine original aging hallmarks; however, occasionally, we inferred a particular taxonomy term that was not specifically stated in original paper (Figure 1, Table S1). Additional taxonomy levels represented increasingly specific biological processes within a subclass (Table S1). Synonyms for each aging hallmark taxonomy term were retrieved from the Unified Medical Language System (UMLS) Metathesaurus (Bodenreider, 2004) from the U.S. National Library of Medicine (NLM) and relevant review articles. The aging hallmark taxonomy term synonyms were combined to form an aging hallmark dictionary and then linked to the corresponding original aging hallmarks.

#### 4.2.2 Age‐related disease dictionary

The ARD definition was developed previously by applying a hierarchical agglomerative clustering algorithm to clinical data on 278 diseases (Kuan et al., 2021). Four of nine “main” clusters contained 207 diseases, and these diseases also had an adjusted *R* ^2^ of greater than 0.85 on the Gompertz–Makeham model (Kuan et al., 2021). These 207 diseases were defined as ARDs (Table S2) (Kuan et al., 2021). Four ARDs that did not translate effectively to scientific text mining approaches were eliminated from further analysis (Table S2). We next retrieved synonyms for each of the remaining 203 ARDs from the MeSH thesaurus from the NLM (NCBI Resource Coordinators, 2017). The Comparative Toxicogenomics Database's “merged disease vocabulary” (Davis et al., 2019) was downloaded on March 21, 2019. It contains the MeSH diseases hierarchy processed in a CSV file. Supplementary concepts and animal diseases were excluded. This left 4789 human diseases mapped to 28,638 entry terms, or synonyms, after processing. MeSH terms were assigned to the 188 of 203 ARDs from the 4789 diseases. The 188 ARDs were mapped to a hierarchical tree of 1427 rows containing MeSH term subclasses of assigned MeSH terms, of which, 545 relevant subclasses were kept. The synonyms to each subclass were edited manually and then merged for each ARD. For the remaining 15 ARDs, synonyms were derived from the Unified Medical Language System (UMLS) Metathesaurus (Bodenreider, 2004). The synonyms were merged to form an ARD dictionary and then linked to the corresponding 203 ARDs.

#### 4.2.3 Calculating the Ochiai coefficient

The aging hallmark dictionary and human ARD dictionary were matched against the 20.48 million sentences from PubMed titles and abstracts. About 19 ARDs with <250 associated sentences were eliminated (Table S2). The co‐occurrence of the nine aging hallmarks with the remaining 184 ARDs was scored at the sentence level using the Ochiai coefficient (Ochiai, 1957) (Equation 1). The Ochiai coefficient (*OC*_(_ *_H_*_,_ *_D_*_)_) adjusts for the fact that certain ARDs are frequently studied in the biomedical literature while others are infrequently studied. For a given aging hallmark and ARD, nHD is the total number of sentences that co‐mention the aging hallmark and ARD. nD and nH are the total number of sentences that mention the ARD and aging hallmark, respectively (Equation 1) (Lage et al., 2008).

(1) $$OC_{\left(H,D\right)}=\sqrt{\frac{nHD^{2}}{nH\cdot nD}}$$

#### 4.2.4 Verifying extracted associations by manual curation

Age‐related diseases and aging hallmarks with higher Ochiai coefficients are likely to be related in some way, but the type of relationship is not known (Jensen et al., 2006). Therefore, we manually assessed the sentences co‐mentioning aging hallmarks and ARDs to determine whether they correctly reported an association between the aging hallmark and ARD (Table S5) (Yang et al., 2016). Our approach to manual curation was to define co‐mentioning sentences as either (1) “confirmed association” where an aging hallmark is reported (or inferred) to have a role in the ARD development or persistence, (2) “no association,” (3) “irrelevant,” or (4) “error” (Gutierrez‐Sacristan et al., 2015) (Table S3). For aging hallmarks with <2500 co‐mentioning sentences, we manually examined all sentences co‐mentioning a given aging hallmark‐ARD pair until we found one sentence that satisfied the criteria of “confirmed association” (Table S3 and S5). For the remaining aging hallmarks, three sentences that satisfied the criteria of “confirmed association” were required (Table S3 & S5). If an aging hallmark‐ARD pair could not be confirmed by a sufficient number of sentences, its Ochiai coefficient was set to zero to increase the reliability of our findings. The 30 highest scoring ARDs were selected for each aging hallmark.

### 4.3 Analysis of aging hallmark‐associated multimorbidity subnetworks and network propagation

#### 4.3.1 Multimorbidity networks

We used multimorbidity networks derived from previously analyzed clinical data on 289 diseases, including the 184 ARDs, in 3.01 million individuals (Kuan, 2020; Kuan et al., 2021). The clinical data were obtained from Clinical Practice Research Datalink (CPRD), which was linked to the Hospital Episode Statistics admitted patient care (HES APC) dataset and accessed via the CALIBER research platform (Kuan, 2020; Kuan et al., 2021). From the multimorbidity network data, we derived an undirected ARD network, where the nodes represent the 184 ARDs which were connected by edges. Edges linked ARD nodes if they were linked by a positive, significant partial correlation (after Bonferroni correction). The partial correlation was used as the edge weight (Kuan, 2020; Kuan et al., 2021). 170 of the 184 ARDs had a median age of first recorded diagnosis 50 years or older (Kuan, 2020; Kuan et al., 2021). Therefore, we used four multimorbidity networks for the 184 ARDs representing age categories from 50 years (Table S6) (Kuan, 2020; Kuan et al., 2021).

#### 4.3.2 Network analysis of top 30 ranked aging hallmark‐associated ARDs

We selected the top 30 ranking nodes for each aging hallmark from each of the four multimorbidity networks and, therefore, plotted 36 subgraphs. The network density (*D*) was calculated for each subnetwork using the algorithm shown in Equation 2 where *E* is the number of edges in a subnetwork and *V* is the number of nodes in a subnetwork.

(2) $$D=\frac{2E}{V\left(V‐1\right)}$$

For each aging hallmark and age category, we shuffled the updated Ochiai coefficient associated with the 184 ARDs 20,000 times. At each shuffle, we selected the top 30 ARD nodes to form a subnetwork and calculated their network density. For a given permutation, each time the random network density (*D_k_*) was greater than or equal to the actual network density (*D*_0_) we added a score of 1, and otherwise 0. The *p*‐value (*p*) for the network density was derived using Equation 3 where *K* is the total number of permutations (Qian et al., 2014).

(3) $$p=\frac{\sum_{k=1}^{K}I\left(D_{k}\geq D_{O}\right)}{K}$$

The *p*‐value was corrected for multiple testing across the 4 age categories per aging hallmark using the Benjamini–Hochberg procedure (Benjamini & Hochberg, 1995).

#### 4.3.3 Network propagation onto multimorbidity networks

For each aging hallmark and age category, the updated Ochiai coefficient scores (*F* ^0^) were superimposed onto each of the ARD nodes of the multimorbidity network. Using a Random Walk with Restart (RWR) algorithm, the scores were smoothed over the network (Equation 4) from the R package BioNetSmooth version 1.0.0 to derive the posterior score (Chokkalingam et al., 2021).

(4) $$F^{i}=\alpha W\prime F^{i‐1}+\left(1‐\alpha \right)F^{0}$$

In the RWR algorithm, *F^i^* and *F^i^* ^−1^ are the posterior evidence of association of an aging hallmark with an ARD at smoothing iteration, *i* and *i*−1, respectively, and we iterated until convergence (*i* = 30). The degree row‐normalized adjacency matrix of the weighted disease network is represented by *W*′. The entries in the adjacency matrix (i.e., $W\prime =[w\prime_{r,c}]$) are defined in Equation 5,

(5) $$w\prime_{r,c}=\left\{\begin{array}{cc} \frac{w_{r,c}}{d_{r}}, & \text{if}\;v_{r}\;\text{is}\;\text{adjacent}\;\text{to}\;v_{c}\\ 0, & \text{otherwise} \end{array}\right.,$$

where *d_r_* is the degree of the ARD node *v_r_* and the edge weight between ARD node *v_r_* and ARD node *v_c_* is *w_r_*_,_ *_c_*. Alpha (*α*) was set at 0.5. The top 30 ARDs with the highest posterior score after network propagation were selected to form a subnetwork. Significant subnetworks were identified using the approach described previously (Equation 3) with correction for multiple testing (Benjamini & Hochberg, 1995). We identified ARDs newly prioritized in the top 30 ARDs associated with an aging hallmark in these subnetworks, which also had an incompletely understood pathogenesis or pathophysiology.

### 4.4 Identification of functionally enriched biological processes using genetic data

#### 4.4.1 Genes underlying ARDs

The NHGRI‐EBI GWAS Catalog (Buniello et al., 2019) was downloaded on February 26, 2020. 103 of the 203 defined ARDs were represented in the GWAS catalog (Buniello et al., 2019). These 103 ARDs were mapped to 181 “Mapped Traits,” which are terms from the Experimental Factor Ontology that are assigned to each GWAS and represent, for example, the disease investigated (Buniello et al., 2019). Single nucleotide polymorphisms (SNPs) with a *p*‐value of <5 × 10^−8^ associating them to ARDs were kept. GWAS studies in European populations were included; however, certain groups were excluded (e.g., Amish). SNPs located were assigned to genes (i.e., Ensembl Gene IDs) if they were located within a gene or intergenic SNPs less than 50 kilobase pairs (kbp) from a gene. For newly prioritized ARDs after network propagation, intergenic SNPs were assigned to genes at a distance of 75 kbp to maximize retrieval of relevant genes. The Ensembl gene IDs were mapped to National Centre for Biotechnology Information (NCBI) Gene IDs, where available, using the NCBI Gene database of *Homo sapiens* (Brown et al., 2015). Thus, 2364 NCBI Gene IDs were linked to 84 ARDs and 135 Mapped Traits subclasses.

#### 4.4.2 Functional enrichment of biological processes for top 30 ARDs mapped to aging hallmarks

We identified the union of genes linked to the top 30 ARDs per aging hallmark (based on text mining) (Figure 2b). The NCBI Gene IDs for protein‐coding genes were mapped to “stringId”s using the STRING database forming nine protein lists (Szklarczyk et al., 2019). Of all 86 ARDs included in top 30 ranked node subnetworks, 55 were associated with 1698 NCBI Gene IDs and mapped to 1693 stringId. The background set was also downloaded from the STRING database on January 27, 2019 (Szklarczyk et al., 2019), which contained 16,598 stringIds mapped to the biological process GO terms. 1560 of 1693 stringIds were also in the background set (Johnson et al., 2015). We used topGO (Alexa et al., 2006) with Fisher's exact test to identify biologically enriched processes against the background set and applied the “weight01” algorithm to reduce redundancy of GO terms. The final *p*‐value cutoff was 0.05, and the minimum node size was 5. Using our previously created aging hallmark dictionary, we searched for GO terms related to the aging hallmarks. Shortened synonyms and abbreviations were appended to the dictionary for specific aging hallmarks. We also searched for GO terms related to “pathway” and “cascade,” and we kept only the pathways that were significantly enriched across all aging hallmark protein lists.

### 4.5 Computational analyses and images

Computational analyses were carried out in Python 3.7.0 and R Version 3.3.0 and 3.6.0. Aging hallmark and ARD images were downloaded from Adobe Stock and Shutterstock after obtaining a standard license.

## CONFLICT OF INTEREST

The authors have no financial conflicts of interest to disclose. At the time of conducting this research, MZ was employed at BenevolentAI. Since completing the work, MZ is now a full‐time employee of GlaxoSmithKline.

## AUTHOR CONTRIBUTIONS

HCF, LP, AB, RJ, and VK involved in conceptualization and design of the study. HCF performed the analysis. HCF, LP, AB, RJ, VK, MZ, ADH, AL, and JG interpreted the data and the results. HCF, LP, AB, VK, MZ, RJ, and ADH drafted the paper and reviewed the drafts.

## Supporting information

Supplementary Material

## Data Availability

The custom code to reproduce the analysis, and the data sets generated and analyzed in this research article are available on request.
