# Supplementary material for: Biological mechanisms of aging predict age‐related disease co‐occurrence in patients
Source: Aging Cell. 2022 Mar 8;21(4):e13524. doi: 10.1111/acel.13524 (PMC9009120; doi:10.1111/acel.13524)
Supplement: Supplementary file 1 — Supplementary Material [file ACEL-21-e13524-s001.pdf]

Supplementary Figures

Figure S1. **P-value of enriched biological processes across all aging hallmarks.** We identified the genes linked to each of the top 30 ARDs associated with an aging hallmark from text mining. We took the union of genes leading to nine gene sets. Protein-coding genes within each gene set were mapped to proteins forming nine protein sets. The associated aging hallmark from text mining represents the column labels (i.e. GI, TA, EA, LOP, CS, DNS, MD, SCE and AIC). We carried out GSEA and expected significant enrichment of GO terms related to the same aging hallmark to verify our findings from text mining. After GSEA, we searched for GO terms related to aging hallmarks: (a) GI, (b) TA, (c) EA, (d) LOP, (e) DNS, (f) MD, (g) CS, (h) SCE and (i) AIC. The 'aging hallmark (AH) GO Term' column highlights the retrieved GO terms linked to each aging hallmark. The abbreviations are listed in Table S9.

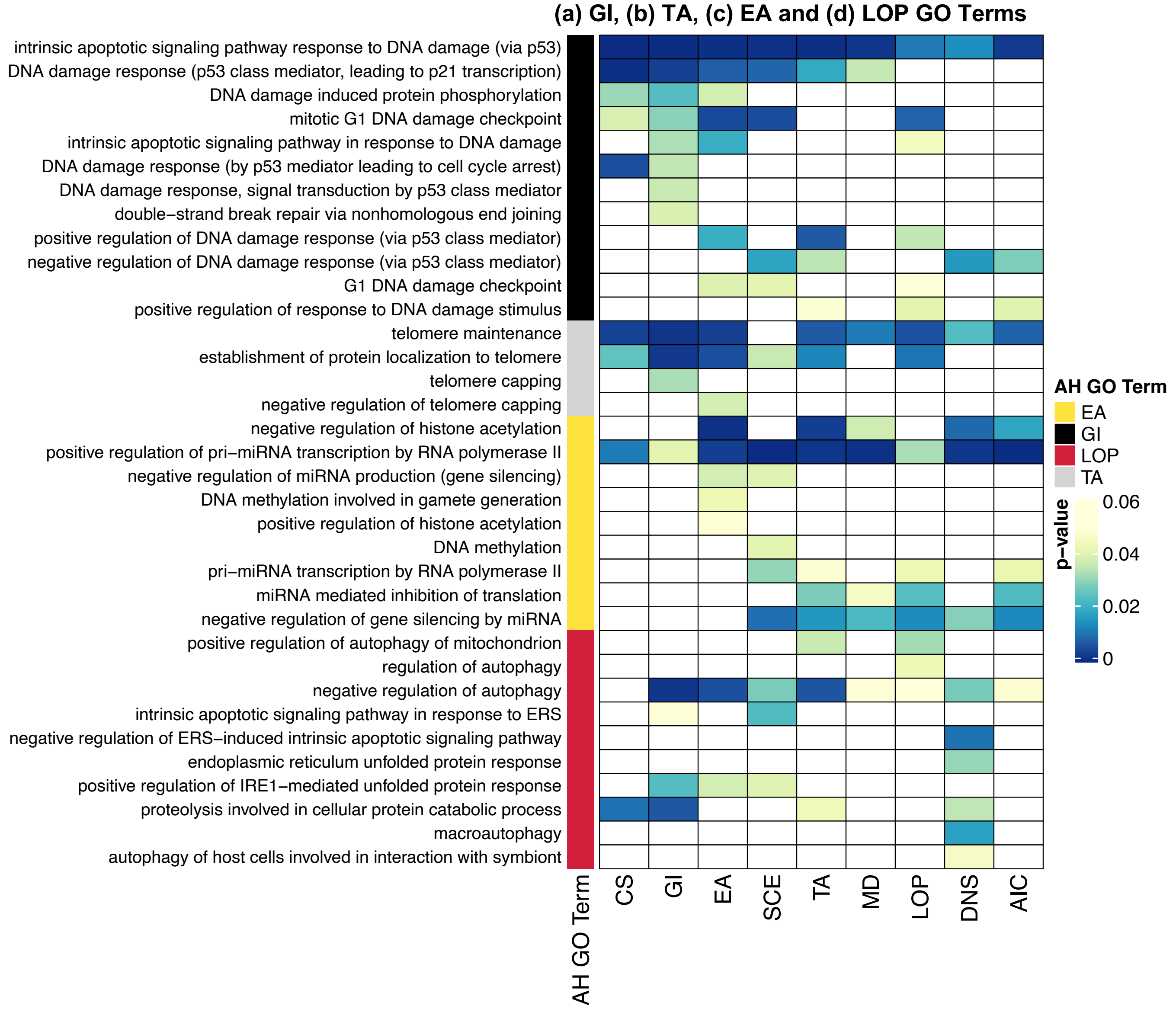

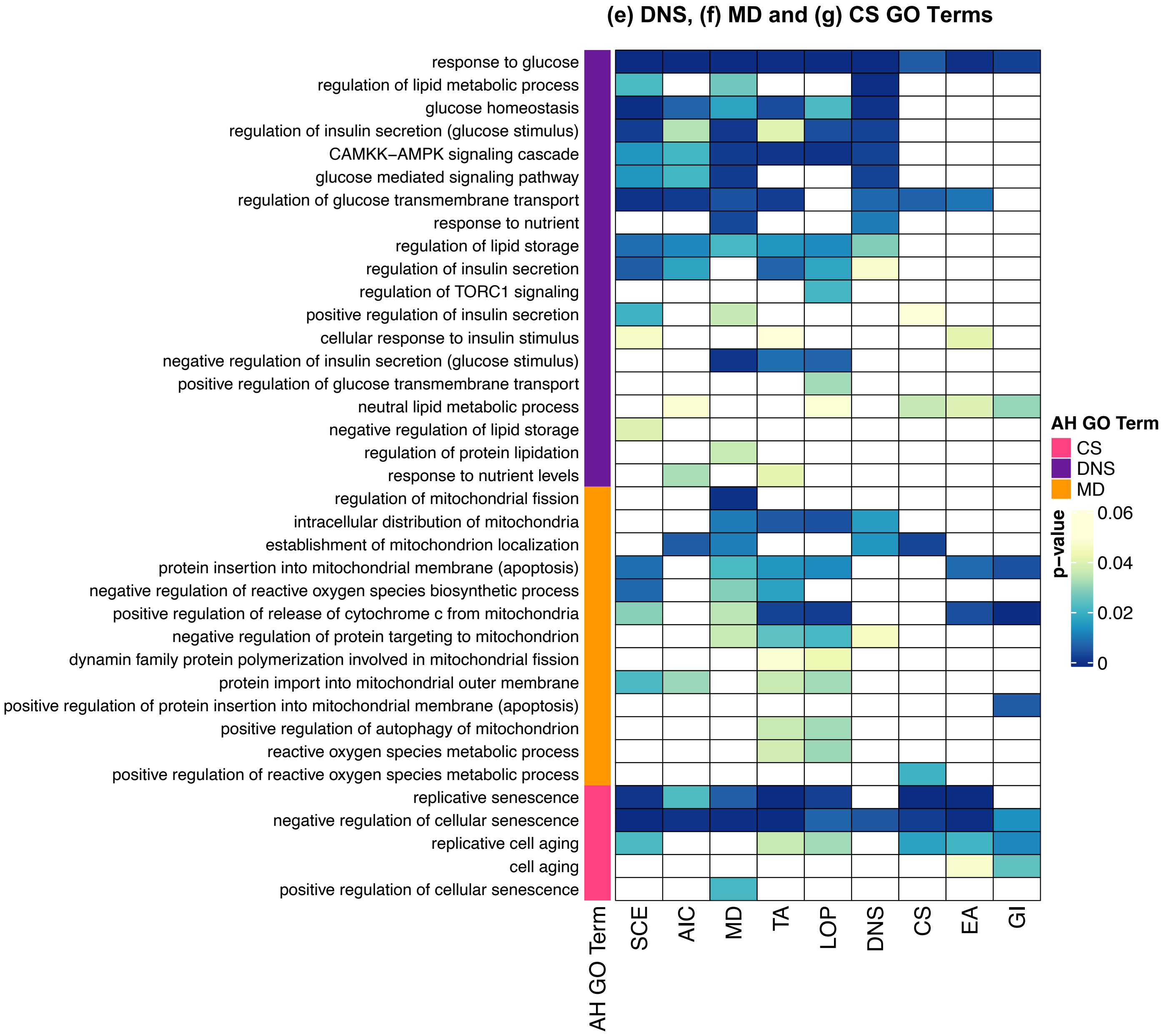

(e) DNS, (f) MD and (g) CS GO Terms
response to glucose
regulation of lipid metabolic process
glucose homeostasis
regulation of insulin secretion (glucose stimulus)
CAMKK−AMPK signaling cascade
glucose mediated signaling pathway
regulation of glucose transmembrane transport
response to nutrient
regulation of lipid storage
regulation of insulin secretion
regulation of TORC1 signaling
positive regulation of insulin secretion
cellular response to insulin stimulus
negative regulation of insulin secretion (glucose stimulus)
positive regulation of glucose transmembrane transport
neutral lipid metabolic process
negative regulation of lipid storage
regulation of protein lipidation
response to nutrient levels
regulation of mitochondrial fission
intracellular distribution of mitochondria
establishment of mitochondrion localization
protein insertion into mitochondrial membrane (apoptosis)
negative regulation of reactive oxygen species biosynthetic process
positive regulation of release of cytochrome c from mitochondria
negative regulation of protein targeting to mitochondrion
dynamin family protein polymerization involved in mitochondrial fission
protein import into mitochondrial outer membrane
positive regulation of protein insertion into mitochondrial membrane (apoptosis)
positive regulation of autophagy of mitochondrion
reactive oxygen species metabolic process
positive regulation of reactive oxygen species metabolic process
replicative senescence
negative regulation of cellular senescence
replicative cell aging
cell aging
positive regulation of cellular senescence
AH GO Term
SCE
AIC
MD
TA
LOP
DNS
CS
EA
GI
AH GO Term
CS
DNS
MD
p−value
0.06
0.04
0.02
0

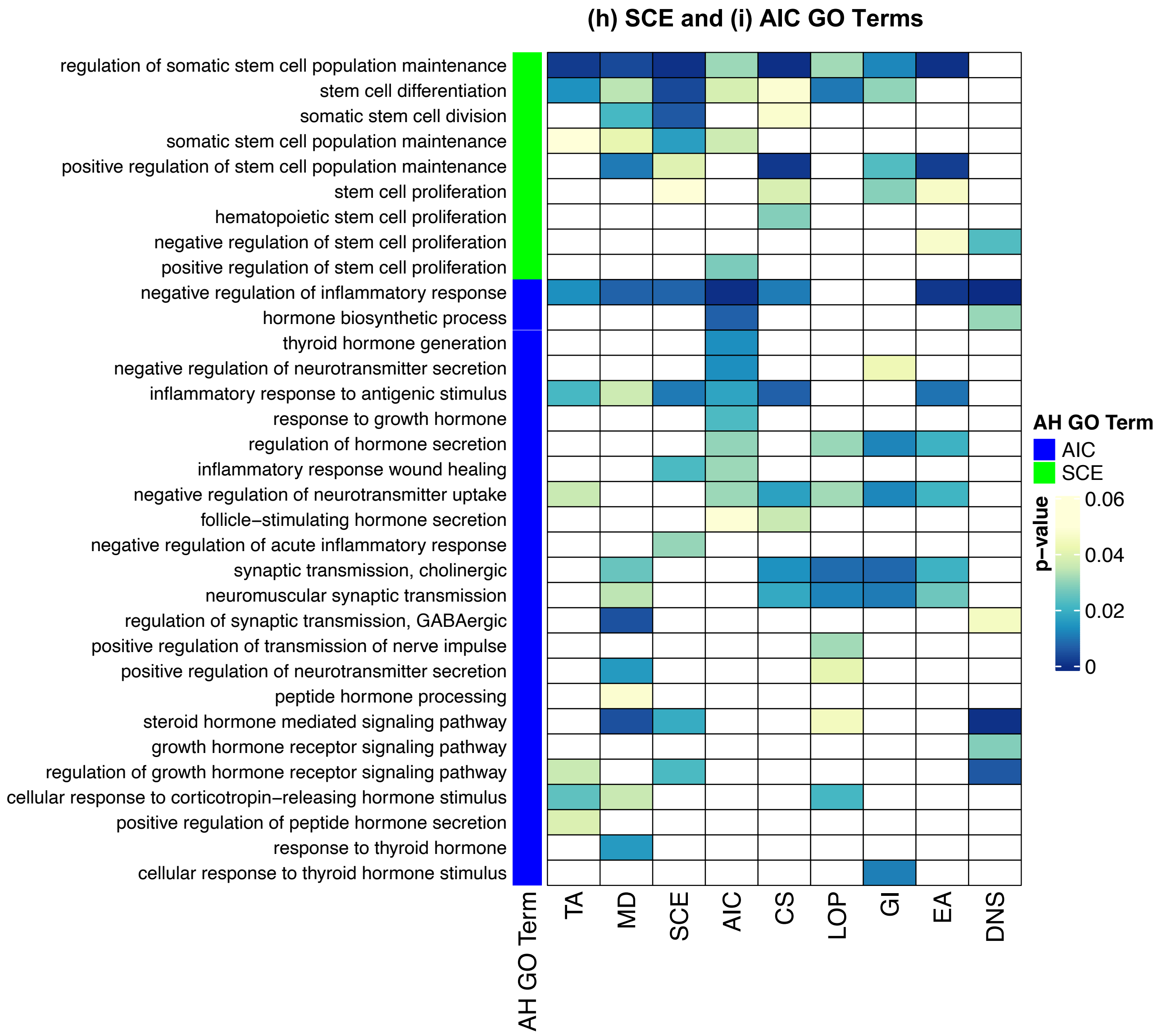

(h) SCE and (i) AIC GO Terms
regulation of somatic stem cell population maintenance
stem cell differentiation
somatic stem cell division
somatic stem cell population maintenance
positive regulation of stem cell population maintenance
stem cell proliferation
hematopoietic stem cell proliferation
negative regulation of stem cell proliferation
positive regulation of stem cell proliferation
negative regulation of inflammatory response
hormone biosynthetic process
thyroid hormone generation
negative regulation of neurotransmitter secretion
inflammatory response to antigenic stimulus
response to growth hormone
regulation of hormone secretion
inflammatory response wound healing
negative regulation of neurotransmitter uptake
follicle−stimulating hormone secretion
negative regulation of acute inflammatory response
synaptic transmission, cholinergic
neuromuscular synaptic transmission
regulation of synaptic transmission, GABAergic
positive regulation of transmission of nerve impulse
positive regulation of neurotransmitter secretion
peptide hormone processing
steroid hormone mediated signaling pathway
growth hormone receptor signaling pathway
regulation of growth hormone receptor signaling pathway
cellular response to corticotropin−releasing hormone stimulus
positive regulation of peptide hormone secretion
response to thyroid hormone
cellular response to thyroid hormone stimulus
AH GO Term
TA
MD
SCE
AIC
CS
LOP
GI
EA
DNS
AH GO Term
AIC
SCE
p−value
0.06
0.04
0.02
0

Figure S2. Subnetworks of top 30 ranked ARDs after network propagation for (a) mitochondrial dysfunction (60-69 years) and (b) deregulated nutrient sensing (60-69 years). Nodes are coloured by ARD ranking after network propagation for a given aging hallmark. The 1st to 10th ranked ARDs for a given aging hallmark are red, the 11th to 20th ranked ARD for a given aging hallmark are in orange and the 21st to 30th ranked ARDs for a given aging hallmark are yellow. The abbreviations are listed in Table S9.

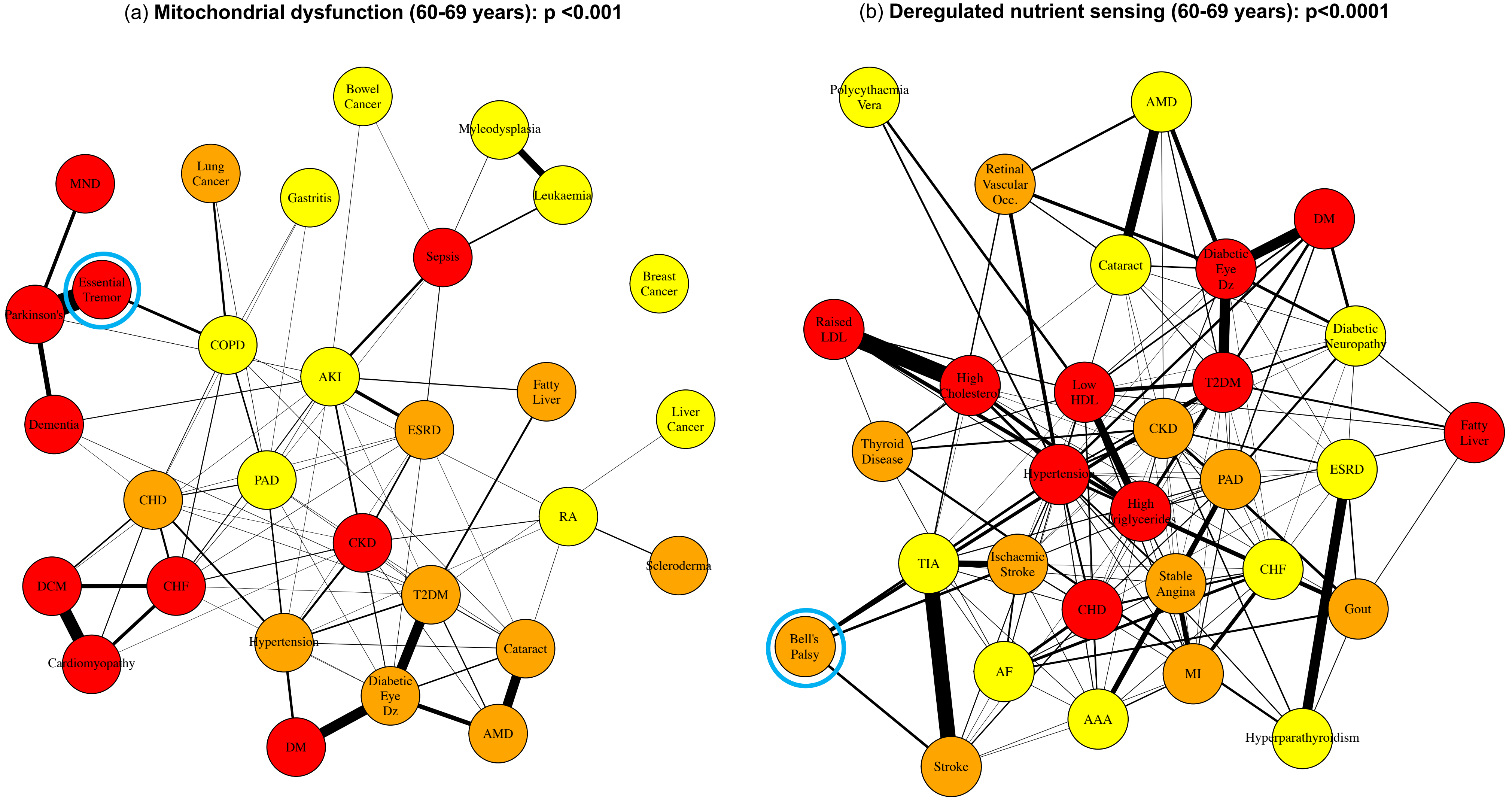

Supplementary Tables

Table S1. The 65 aging hallmark taxonomy terms derived from "*The Hallmarks of Aging*" paper. The table shows quotations from "*The Hallmarks of Aging*" paper by Lopez-Otin *et al*. (2013), which support the selection of taxonomy terms. Occasionally, taxonomy terms are inferred rather than being directly mentioned in "*The Hallmarks of Aging*" , which is indicated with (*i*).

Original Aging Hallmark	Taxonomy Term(s)	Supporting quotations from "*The Hallmarks of Aging*" (Lopez-Otin *et al*., 2013) or rationale
**Genomic Instability**	Genomic instability	"**Genomic Instability**" (Hallmark of Aging)
	Somatic mutations	"**Somatic mutations** accumulate within cells from aged humans."
	DNA damage,<br>Chromosomal breakage	"Other forms of **DNA damage**, such as **chromosomal aneuploidies** and copy number variations, have also been found associated with aging."
	Transposable elements	"The genetic lesions arising from extrinsic or intrinsic damage are highly diverse and include … gene disruption caused by the integration of viruses or **transposons**".
	DNA repair deficiencies	"**Deficiencies in DNA repair mechanisms** cause accelerated aging."
	mtDNA mutations	"**Mutations** and deletions in aged **mtDNA** may also contribute to aging."
	mtDNA damage	"The first evidence that **mtDNA damage** might be important for aging and ARDs derived from the identification of human multisystem disorders caused by mtDNA mutations."
	Double strand (ds)-DNA breaks	"Note that both nuclear DNA and mitochondrial DNA are subjected to age-associated genomic alterations … **double strand breaks**."
	DNA breaks (*i*),<br>Single strand (ss)-DNA breaks (*i*)	"The **genetic lesions** arising from intrinsic and extrinsic damages are highly diverse and include point mutations … ". Additional examples include **single-strand DNA breaks** or, more generally, **DNA breaks**[1].
**Telomere Attrition**	Telomere attrition	"**Telomere Attrition**" (Hallmark of Aging)
	Decreased telomere length,<br>Decreased leukocyte telomere length (*i*)	"In humans, recent meta-analyses have indicated a strong relation between **short telomeres** and mortality risk, particularly at younger ages." **Leukocyte telomere length** is a more specific subcategory of decreased telomere length[2].
**Epigenetic Alterations**	Epigenetic alterations	"**Epigenetic Alterations**" (Hallmark of Aging)
	Gene transcription,<br>coding-RNAs	"Aging is associated with an increase in **transcriptional** noise, and an **aberrant production and maturation of many mRNAs**."
	microRNAs,<br>non-coding RNAs	"The aging-associated transcriptional signatures also affect **non-coding RNAs**, including a class of **miRNAs** that is associated with the aging process".
	Histone modifications,<br>histone acetylation,<br>histone methylation,<br>DNA methylation	"Alterations in the **acetylation** and **methylation** of **DNA** or **histones**, as well as of other chromatin-associated proteins, can induce epigenetic changes that contribute to the aging process."

**Loss of Proteostasis**	Loss of proteostasis	"**Loss of Proteostasis**" (Hallmark of Aging)
	Proteolysis,<br>autophagy,<br>proteasome	"The activities of the two principal **proteolytic** systems implicated in protein quality control, namely, the **autophagy-lysosomal** system and the **ubiquitin-proteasome system**, decline with aging."
	Protein aggregation	"Failure to refold or degrade unfolded proteins can lead to their accumulation and **aggregation**, resulting in proteotoxic effects."
	ER stress,<br>unfolded protein response (UPR)	"Endogenous and exogenous stress causes the unfolding of proteins … **ER Stress. Unfolded proteins** are usually refolded by heat-shock proteins (HSP) or targeted to destruction by the ubiquitin-proteasome or lysosomal (autophagic) pathways."
	Chaperone	"The stress-induced synthesis of cytosolic and organelle-specific **chaperones** is significantly impaired in aging."
**Deregulated Nutrient Sensing**	Deregulated nutrient sensing	"**Deregulated Nutrient Sensing**" (Hallmark of Aging)
	Insulin resistance,<br>dyslipidaemia *(i)*	"Thus, a **decreased IIS** is a common characteristic of both physiological and accelerated aging, while a constitutively decreased IIS extends longevity." **Dyslipidaemia** is also associated with deregulated nutrient sensing[3].
	IIS pathway,<br>nutrient-sensing pathways	"In addition to the **IIS pathway** that participates in glucose-sensing, three additional related and interconnected **nutrient-sensing systems** are the focus of intense investigation…"
	mTORC1	"These observations, together with those involving the IIS pathway, indicate that intense trophic and anabolic activity, signalled through the IIS or the **mTORC1** pathways, are major accelerators of aging."
	AMPK,<br>sirtuin 1	"The other two nutrient sensors, **AMPK** and **sirtuins**, act in the opposite direction to IIS and mTOR, meaning that they signal nutrient scarcity and catabolism instead of nutrient abundance and anabolism. Accordingly, their up-regulation favours healthy aging. Moreover, **SIRT1** and **AMPK** can engage in a positive feedback loop, thus connecting both sensors of low-energy states into a unified response."
**Mitochondrial Dysfunction**	Mitochondrial dysfunction	"**Mitochondrial Dysfunction**" (Hallmark of Aging)
	Mitochondrial toxicity *(i)*	"Mitochondrial function becomes perturbed by aging-associated mtDNA mutations …". Another cause of perturbed mitochondrial function is toxin exposure, such as from alcohol, termed **mitochondrial toxicity**[4].
	Reactive oxygen species (ROS)	"As chronological age advances, the levels of **ROS** increase in an attempt to maintain survival until they betray their original purpose and eventually aggravate, rather than alleviate, the age-associated damage."

	Mitochondrial bioenergetics, mitochondrial biogenesis	"The reduced efficiency of **mitochondrial bioenergetics** with aging may result from multiple converging mechanisms including reduced **biogenesis of mitochondria**."
	Mitochondrial turnover, mitochondrial degradation	"The combination of increased damage and **reduced turnover in mitochondria**, due to lower biogenesis and **reduced clearance**, may contribute to the aging process."
	Electron transport chain (ETC), mitochondrial dynamics, Krebs cycle *(i)*	"Mitochondrial function becomes perturbed by aging-associated mtDNA mutations, reduced mitochondriogenesis, destabilization of the **electron transport chain (ETC)** complexes, altered **mitochondrial dynamics** or defective quality control by mitophagy." The **Krebs cycle** produces electron carriers that pass electrons to the electron transport chain (ETC).
**Cellular Senescence**	Cellular senescence	"**Cellular Senescence**" (Hallmark of Aging)
	Senescence markers	"The accumulation of senescent cells in aged tissues has been often inferred using **surrogate markers**, such as DNA damage. Some studies have directly used senescence-associated β-galactosidase (SABG) to identify senescence in tissues."
	Senescence-associated secretory phenotype (SASP)	"Senescent cells manifest dramatic alterations in their secretome, which … is referred to as the '**senescence-associated secretory phenotype'**."
	Immunosenescence (*i*)	Immunosenescence describes age-related alterations in the function of the immune system[5] and is partly explained by cellular senescence[6].
**Stem Cell Exhaustion**	Stem cell exhaustion	"**Stem Cell Exhaustion**" (Hallmark of Aging)
	Progenitor cell, stem cell	"Although deficient proliferation of **stem** and **progenitor cells** is obviously detrimental for the long-term maintenance of the organism."
	Stem cell self-renewal	"Studies on aged mice have revealed an overall decrease in cell cycle activity of hematopoietic **stem cells** (HSCs), with old HSCs undergoing **fewer cell divisions** than young HSCs."
**Altered Intercellular Comm.**	Altered intercellular communication	"**Altered Intercellular Communication**" (Hallmark of Aging)
	Endocrine signalling, neuronal signalling, hormone *(i)*, neurotransmitters *(i)*	"Aging also involves changes at the level of intercellular communication, be it **endocrine**, **neuroendocrine** or **neuronal**." In endocrine signalling, the signalling molecules are **hormones.** In neuronal signalling, the signalling molecules **neurotransmitters**.
	Inflammaging	"A prominent aging-associated alteration in intercellular communication is '**inflammaging'**."
	Inflammatory signalling	"Inflammaging may result from multiple causes such as the accumulation of **pro-inflammatory** tissue damage."
	Inflammation	"**Inflammation**" (Subheading)

Table S2. The original list of 207 ARDs, of which, 184 ARDs were included in the analysis. The 23 ARDs that were excluded from further analysis are shown in italics including: (i) 4 ARDs that were not specific enough for scientific literature mining (*****) and (ii) 19 ARDs with less than 250 associated publications (•). ARDs are categorised for clarity.

ARD Category	List of ARDs
**Cancers**	Adrenal metastases
	Biliary cancer
	Bladder cancer
	Bone cancer
	Bone metastases
	Bowel cancer
	Bowel metastases•
	Brain cancer
	Brain metastases
	Breast cancer
	Kidney cancer
	Leukaemia
	Liver cancer
	Liver metastases
	Lung cancer
	Lung metastases
	Lymph node metastases
	Melanoma
	Mesothelioma
	Monoclonal gammopathy of undetermined significance (MGUS)
	Myelodysplasia
	Non-Hodgkin lymphoma (NHL)
	Oesophageal cancer
	Oropharyngeal cancer
	Ovarian cancer
	Pancreatic cancer
	Peritoneal metastases
	Plasma cell cancer
	Pleural metastases
	Polycythaemia vera
	*Primary malignancy (other)**
	Prostate cancer
	*Secondary malignancy (other)**
	Skin cancer
	Stomach cancer
	Thyroid cancer
	Uterine cancer
**Cardiovascular**	Abdominal aortic aneurysm (AAA)
	Atrial fibrillation (AF)
	Atrioventricular (AV) block (third degree)
	Atrioventricular block (first degree)•
	Atrioventricular block (second degree)•
	Bifascicular block•
	Cardiomyopathy
	Coronary heart disease (CHD)
	Dilated cardiomyopathy (DCM)
	Heart failure (CHF)
	Hypertension
	Hypertrophic cardiomyopathy (HCM)
	Intracerebral haemorrhage

	Ischaemic stroke<br>Left bundle branch block (LBBB)<br>*Multiple valve disorder•*<br>Myocardial infarction (MI)<br>Non-rheumatic aortic valve disorder<br>Non-rheumatic mitral valve disorder<br>Pericardial effusion<br>Peripheral arterial disease (PAD)<br>Primary pulmonary hypertension<br>Pulmonary embolism<br>Raynaud's disease<br>Rheumatic valve disorder<br>Right bundle branch block (RBBB)<br>*Secondary pulmonary hypertension•*<br>Sick sinus syndrome<br>Stable angina<br>Stroke<br>Subarachnoid haemorrhage (SAH)<br>Subdural haematoma<br>Supraventricular tachycardia (SVT)<br>Transient ischaemic attack (TIA)<br>*Trifascicular block•*<br>Unstable angina<br>Venous thromboembolism (VTE)<br>Ventricular tachycardia (VT)
**Benign Neoplasm**	*Benign brain neoplasms•*<br>*Benign colonic neoplasms•*<br>*Benign stomach neoplasms•*
**Digestive**	Abdominal hernia<br>*Angiodysplasia of colon•*<br>Anorectal prolapse<br>Autoimmune liver disease<br>Barrett's oesophagus<br>Cholangitis<br>Cholecystitis<br>Cholelithiasis<br>Cirrhosis<br>Diaphragmatic hernia<br>Diverticular disease<br>Fatty liver<br>Gastritis<br>Gastro-oesophageal reflux disease (GORD)<br>Liver failure<br>*Oesophageal ulcer•*<br>Oesophageal varices<br>Pancreatitis<br>Peptic ulcer<br>Peritonitis<br>Portal hypertension<br>Volvulus
**Endocrine**	Diabetes Mellitus (DM)<br>High (total) cholesterol<br>High triglycerides<br>Hyperparathyroidism

	Low high density lipoprotein cholesterol (low HDL)<br>Raised low density lipoprotein cholesterol (raised LDL)<br>Syndrome of inappropriate anti-diuretic hormone (SIADH)<br>Thyroid disease<br>Type 2 diabetes mellitus (T2DM)
**Ear**	Deafness<br>Meniere's disease<br>Tinnitus
**Eye**	Anterior uveitis<br>Blindness<br>Cataract<br>Diabetic eye disease<br>Glaucoma<br>Keratitis<br>Macular degeneration (AMD)<br>Ptosis<br>Retinal detachment<br>Retinal vascular occlusion
**Genitourinary**	Acute kidney injury (AKI)<br>Benign prostatic hyperplasia (BPH)<br>*Chronic cystitis•*<br>Chronic kidney disease (CKD)<br>End stage renal disease (ESRD)<br>Glomerulonephritis<br>Hydrocele<br>Neuropathic bladder<br>Obstructive and reflux uropathy<br>Tubulo-interstitial nephropathy<br>Urinary incontinence<br>Uterovaginal prolapse
**Haematological/ Immunological**	Agranulocytosis<br>Anaemia<br>Aplastic anaemia<br>*Folate deficiency anaemia•*<br>Haemolytic anaemia<br>Hypersplenism<br>*Hyposplenism•*<br>Immunodeficiency<br>Iron deficiency anaemia<br>Primary thrombocytopaenia<br>*Secondary polycythaemia•*<br>*Secondary thrombocytopaenia•*<br>*Vitamin B12 deficiency anaemia•*
**Infections**	Bacterial infection (ID)<br>Bone ID<br>Eye infection<br>Fungal infection<br>Gastroenteritis<br>Genitourinary infection (male)<br>Heart infection<br>*Infection of other organs**<br>*Infection with other organisms**<br>Lower respiratory tract infection (LRTI)<br>Nervous system infection

	Parasitic infection<br>Rheumatic fever<br>Sepsis<br>Skin infection<br>Urinary tract infection (UTI)<br>Viral infection
**Musculoskeletal**	Carpal tunnel syndrome<br>Collapsed vertebra<br>Fibromatosis<br>Giant cell arteritis<br>Gout<br>Hip fracture<br>Osteoarthritis<br>Osteoporosis<br>Polymyalgia rheumatica<br>Rheumatoid arthritis (RA)<br>Scleroderma<br>Scoliosis<br>Sjögren’s Syndrome<br>Spinal stenosis<br>Spondylolisthesis<br>Spondylosis<br>Wrist fracture
**Neurological**	Autonomic neuropathy<br>Bell's palsy<br>Diabetic neuropathy<br>Epilepsy<br>Essential tremor<br>Motor neurone disease (MND)<br>Myasthenia gravis<br>Parkinson's disease<br>Peripheral neuropathy<br>Trigeminal neuralgia
**Psychiatric**	Delirium<br>Dementia
**Respiratory**	Asbestosis<br>Aspiration pneumonitis<br>Bronchiectasis<br>Chronic obstructive pulmonary disease (COPD)<br>Pleural effusion<br>*Pleural plaque*•<br>Pneumothorax<br>Pulmonary collapse<br>Pulmonary fibrosis<br>Respiratory failure
**Skin**	Actinic keratosis<br>Dermatitis<br>Lichen planus<br>Seborrheic dermatitis

Table S3. Examples of criteria for defining a sentence as a "confirmed association" between an aging hallmark and an ARD. For example, if a sentence mentions that an ARD is: (i) caused or partially caused by, (ii) associated with, (iii) exacerbated by, or (iv) results in one of the criteria in the table below then a sentence is defined as "*confirmed association*" between an aging hallmark and ARD.

Aging Hallmark	Criteria
Genomic Instability	• DNA damage or injury (e.g. DNA breaks)<br>• mitochondrial DNA damage or injury<br>• single or multiple somatic mutations<br>• genomic instability<br>• genetic alteration(s)
Telomere Attrition	• short or shorter leukocyte telomere length<br>• short or shorter telomere length<br>• telomere attrition, dysfunction or erosion<br>• telomere disorder
Epigenetic Alterations	• epigenetic alterations<br>• changes in DNA methylation<br>• changes in histone acetylation
Loss of Proteostasis	• proteasome dysfunction, dysregulation, failure or impairment<br>• aggregated or misfolded proteins<br>• induction of, or increased endoplasmic reticulum (ER) stress<br>• activation of, or aberrant unfolded protein response (UPR)<br>• inadequate, dysregulated or defective autophagy<br>• impaired proteostasis<br>• impaired chaperone activity<br>• reduced proteolytic activity
Deregulated Nutrient Sensing	• increased insulin resistance<br>• dyslipidaemia<br>• decreased 5' AMP-activated protein kinase (AMPK) activity<br>• decreased sirtuin 1 activity
Mitochondrial Dysfunction	• generation or presence of reactive oxygen species (ROS) or active oxygen<br>• mitochondrial dysfunction or degeneration<br>• altered mitochondrial dynamics or bioenergetics<br>• mitochondrial damage<br>• impaired mitochondrial turnover including mitochondrial biogenesis or mitophagy
Cellular Senescence	• accelerated, early or enhanced cellular senescence, replicative senescence or immunosenescence<br>• activation of cellular senescence or cellular aging<br>• association with the senescence-associated secretory phenotype (SASP)<br>• increased senescent cells or expression of senescence markers
Stem Cell Exhaustion	• reduced number, impaired proliferative capacity or increased destruction of stem cells or progenitor cells<br>• impaired function, mobilization or exhaustion of stem cells or progenitor cells<br>• aging or senescence of stem cells or progenitor cells<br>• decreased number of circulating stem cells or progenitor cells<br>• decreased stem cell or progenitor cell differentiation<br>• stem cell disorder
Altered Intercellular Communication	• increased inflammation<br>• decreased levels of specific hormones, e.g. oestradiol, testosterone<br>• decreased synaptic transmission<br>• increased levels of specific hormones, e.g. parathyroid hormone

Table S4. Examples of sentences correctly reporting that an aging hallmark (yellow) has a role in the development or disordered physiology of an ARD (grey). Sentences such as these were labelled as a "confirmed association" between an aging hallmark and an ARD on manual curation. Those aging hallmark-ARD combinations with insufficient evidence were set to zero. Table S9 provides a list of abbreviations.

Aging Hallmark	ARD (MeSH ID)	PubMed ID	Sentence
GI (DNA damage)	Motor neurone disease (D016472, D000690, D010244)	18344116	"Increased mitochondrial oxidative damage and oxidative DNA damage contributes to the neurodegenerative process in sporadic amyotrophic lateral sclerosis."[7]
TA (Decreased telomere length)	Aplastic anaemia (D000741, D029502, D005199, D029503)	22687638	"Telomere shortening, a well-known marker of aging and cellular stress, occurs under several conditions in the hematopoietic compartment, including aplastic anemia and following iatrogenic noxae."[8]
EA (Epigenetic alterations)	Barrett's oesophagus (D001471)	29046735	"Identification of a key role of widespread epigenetic drift in Barretts oesophagus and oesophageal adenocarcinoma."[9]
LOP (Protein aggregation)	Cataract (D058442, D002386)	23179275	"Cataract, the loss of transparency of eye lens, is a disease of protein aggregation."[10]
DNS (Insulin resistance)	Type 2 diabetes mellitus (D003924)	15579187	"Insulin resistance is the central defect in the development of type 2 diabetes, preceding its onset by 10-20 years."[11]
MD (Reactive oxygen species)	Dementia (D003704, D000544, D015140, D015161)	20933077	"Mitochondrial oxidative stress induced by reactive oxygen species (ROS) has been strongly associated with the pathogenesis of neurodegenerative disorders, including Alzheimer's disease (AD)."[12]
CS (Cellular senescence)	Diabetic retinopathy (D003930)	25162034	"Accumulating evidence has shown that diabetes accelerates aging and endothelial cell senescence is involved in the pathogenesis of diabetic vascular complications, including diabetic retinopathy."[13]
SCE (Progenitor cell)	Osteoarthritis (D010003, D015207, D020370, D055013)	21815581	"The lower chondrogenic and spontaneous osteogenic differentiation of mesenchymal progenitor cells derived from elderly patients may be associated with the development of primary osteoarthritis."[14]
AIC (Inflammation)	Myocardial infarction (D009203, D056988, D056989, D000072658, D0000726657)	10347344	"Inflammation plays a critical role in acute myocardial infarction (AMI) and tumor necrosis factor alpha (TNF-alpha) is a potent inflammatory trigger."[15]

Table S5. **Summary of literature on each aging hallmark in the human aging corpus**. The number of (a) abstracts and (b) sentences mentioning each aging hallmark. (c) The number of sentences also mentioning ARDs per aging hallmark (i.e. co-mentioning the aging hallmark and any ARD).

Aging hallmark	(a) Number of abstracts	(b) Number of sentences	(c) Number of sentences with co-mentions
Genomic Instability	7,313	12,975	2,166
Telomere Attrition	1,823	6,669	864
Epigenetic Alterations	10,751	34,403	8,333
Loss of Proteostasis	5,748	13,915	1,694
Mitochondrial Dysfunction	5,737	11,785	1,257
Deregulated Nutrient Sensing	15,753	34,227	12,682
Cellular Senescence	4,896	10,173	511
Stem Cell Exhaustion	7,466	15,068	2,344
Altered Intercellular Comm.	77,783	133,733	25,958

Table S6. **Features of the ARD multimorbidity networks.** The table provides a summary of the 4 ARD multimorbidity networks including the number of nodes and average network density. We derived subnetworks of the top 30 ARDs for each aging hallmark from these multimorbidity networks.

Age category	Number of ARD nodes	Average network density
50 – 59 years	184	0.0999
60 – 69 years	184	0.0976
70 – 79 years	184	0.0836
≥80 years	184	0.0684

Table S7. Network density of subnetworks of the top 30 ranking ARD nodes after network propagation for age categories 50-59 years, 60-69 years, 70-79 years and ≥80 years. The number of times the network density from permutations (n = 20,000) was greater than or equal to the true network density for that aging hallmark was used to calculate the p-value. The p-value was corrected for multiple testing across the 4 age categories per aging hallmark using the Benjamini-Hochberg procedure (*p < 0.05, ** p <0.01, *** p <0.001, ****p<0.0001).

Aging Hallmark	ARD Network Density			
	50-59 years	60-69 years	70-79 years	≥80 years
Genomic Instability	0.1425	0.1402	0.1310	0.1241
Telomere Attrition	0.1195	0.1356	0.1195	0.1034
Epigenetic Alterations	0.2000**	0.1885**	0.2092***	0.1885***
Loss of Proteostasis	0.0897	0.0805	0.0759	0.0621
Deregulated Nutrient Sensing	0.3448****	0.3632****	0.3448****	0.3034****
Mitochondrial Dysfunction	0.1931****	0.1931***	0.1425***	0.1057**
Cellular Senescence	0.1563	0.1609	0.1379	0.1034
Stem Cell Exhaustion	0.2322****	0.2322***	0.2161***	0.1931***
Altered Intercellular Comm.	0.2207***	0.2161***	0.1977***	0.1540**

Table S8. (a) Inclusion and (b) exclusion criteria were applied when defining the "human aging corpus" to increase relevance of the selected abstracts.

a) Inclusion Criteria	Rationale
Journal articles	Articles were required to be indexed with the "journal article" publication type.
Publication date	Journal articles published between the date of the landmark paper on lifespan extension, "*A C. elegans mutant lives twice as long as wildtype*", on 2nd December 1993 to 31st December 2017 were included.
English language	Abstracts were required to be in English.
Humans	We confined our search to the literature on humans because certain mechanisms of aging are only found in particular evolutionary lineages[16]. Therefore, articles were required to be indexed with the humans Medical Subject Heading (MeSH) term, as certain mechanisms of aging are private.
Aging synonyms	Articles were required to be associated with one of 43 aging terms.
**b) Exclusion Criteria**	**Rationale**
Specific publication types	Reviews, introductory journal articles, retractions of publication, letters to the editor and comments were excluded in order to retrieve high quality original research rather than data from secondary sources. We excluded review articles to avoid re-using content within the scientific literature.
Germline mutation	Abstracts mentioning, or indexed with, 'germline mutations' or related terms were excluded. Germline mutations can be a consequence of the maternal or paternal aging process. Thus, germline mutations were excluded to maximize retrieval of articles focused on the effects of the individual's own aging process.
Telomerase activation and inhibition	Abstracts mentioning 'telomerase activation', 'telomerase inhibition' or related terms were excluded. Telomerase activation is a consequence of cancer progression, as opposed to aging, and leads to telomere lengthening. Therefore, telomerase activation and inhibition were excluded to reduce selection of irrelevant abstracts under the telomere attrition aging hallmark.
Adverse drug effects	Abstracts reporting adverse drug effects, such as DNA damage from chemotherapy for cancer, were excluded to reduce selection of irrelevant abstracts.
Hormone preparations and anti-inflammatory drugs	Abstracts mentioning 'hormone preparations', 'anti-inflammatory drugs' or related terms, were excluded to reduce selection of irrelevant abstracts under the altered intercellular communication aging hallmark.
Stem cell transplant	Abstracts mentioning 'stem cell transplant', or related terms, were excluded to reduce selection of irrelevant abstracts under the stem cell exhaustion aging hallmark.
Induced Pluripotent stem cells (iPSC)	Abstracts mentioning, or indexed with, induced pluripotent stem cells (iPSC) or related terms were excluded. iPSC are used experimentally to treat ARDs, which can result in incorrect selection of abstracts under the stem cell exhaustion aging hallmark.
Neoplastic stem cells	Abstracts mentioning, or indexed with, 'neoplastic stem cells' or related terms were excluded. According to the cancer stem-cell hypothesis, neoplastic stem cells are responsible for cancer tumour growth. This contrasts with stem cell exhaustion, where loss of stem cells leads to ARD development. Therefore, neoplastic stem cells were excluded to avoid incorrect selection of abstracts under the stem cell exhaustion aging hallmark.
GWAS catalog	We excluded PMIDs that were linked to studies included from the GWAS catalog. Thus, the genetic approach to verifying aging hallmark-ARD associations was independent of the literature-based method.

Table S9. Table of abbreviations. Abbreviations are sorted in alphabetical order for (a) aging hallmarks, (b) age-related diseases and (c) genes & proteins.

Abbreviation	Term
(a) Aging hallmark abbreviations	
AH	Aging hallmark
AIC/ Altered intercellular comm.	Altered intercellular communication
AMPK	5’ AMP-activated protein kinase
CS	Cellular senescence
DNA	Deoxyribonucleic acid
DNS	Deregulated nutrient sensing
ds-DNA	Double stranded DNA
EA	Epigenetic alterations
ER	Endoplasmic reticulum
GI	Genomic instability
IIS pathway	Insulin/insulin like growth factor (IGF)-1 signalling pathway
LOP	Loss of proteostasis
LTL	Leukocyte telomere length
MD	Mitochondrial dysfunction
mtDNA	Mitochondrial DNA
mTORC1	mammalian Target Of Rapamycin Complex 1
RNA	Ribonucleic acid
ROS	Reactive oxygen species
SASP	Senescence associated secretory phenotype
SCE	Stem cell exhaustion
ss-DNA	Single stranded DNA
TA	Telomere attrition
TL	Telomere length
UPR	Unfolded protein response
(b) Age-related disease abbreviations	
AAA	Abdominal aortic aneurysm
AF	Atrial fibrillation
AKI	Acute kidney injury
AMD	Age-related macular degeneration
ARD	Age-related disease
Autoimmune liver Dz.	Autoimmune liver disease
Bacterial ID	Infectious disease, bacterial
Barrett’s	Barrett’s oesophagus
Bone ID	Infectious disease of the bone
BPH	Benign prostatic hyperplasia
CHD	Coronary heart disease
CHF	Congestive heart failure
CKD	Chronic kidney disease
COPD	Chronic obstructive pulmonary disease
DCM	Dilated cardiomyopathy
Diabetic Eye Dz.	Diabetic eye disease/ diabetic retinopathy
DM	Diabetes mellitus
ESRD	End stage renal disease
GORD	Gastro-oesophageal reflux disease
HCM	Hypertrophic cardiomyopathy
Low HDL	Low high-density lipoprotein cholesterol
LRTI	Lower respiratory tract infection
Mets.	Metastases

MGUS	Monoclonal gammopathy of undetermined significance
MI	Myocardial infarction
MND	Motor neurone disease
NHL	Non-Hodgkin’s lymphoma
PAD	Peripheral arterial disease
Parkinson’s	Parkinson’s disease
Pri. thrombocytopaenia	Primary thrombocytopaenia
RA	Rheumatoid arthritis
Raised LDL	Raised low density lipoprotein (LDL) cholesterol
Retinal vascular occ.	Retinal vascular occlusion
SAH	Subarachnoid haemorrhage
SIADH	Syndrome of inappropriate anti-diuretic hormone (ADH)
Sjogren’s	Sjogren’s syndrome
T2DM	Type 2 diabetes mellitus
TIA	Transient ischaemic attack
UTI	Urinary tract infection
VTE	Venous thromboembolism
(c) Gene & protein abbreviations	
ABCA7	ATP binding cassette subfamily A member 7
AGER	Advanced glycosylation end-product specific receptor
ANGPT1	Angiopoietin 1
ATAD5	ATPase family AAA domain containing 5
BAG6	BAG cochaperone 6
BCAR1	BCAR1 scaffold protein, Cas family member
BCL3	BCL3 transcription coactivator
BRAF	B-Raf proto-oncogene, serine/threonine kinase
BRCA2	BRCA2 DNA repair associated
BTN3A1	Butyrophilin subfamily 3 member A1
CAMK2(B/D/G)	Calcium/calmodulin dependent protein kinase II (beta/ delta/ gamma)
CD(28/36/247/226)	CD28/ CD36/ CD247/ CD226 molecule
CDKN1A	Cyclin dependent kinase inhibitor 1A
CFLAR	CASP8 and FADD like apoptosis regulator
CHEK2	Checkpoint kinase 2
CHUK	Component of inhibitor of nuclear factor kappa B kinase complex
CTNNA3	Catenin alpha 3
DENND1B	DENN domain containing 1B
DSTYK	Dual serine/threonine and tyrosine protein kinase
ERK1/2	Mitogen-activated protein kinase 3/ 1
FBXW7	F-box and WD repeat domain containing 7
FFAR4	Free fatty acid receptor 4
FGA	Fibrinogen alpha chain
FGB	Fibrinogen beta chain
FGF10	Fibroblast growth factor 10
FGFR2	Fibroblast growth factor receptor 2
FGFR3	Fibroblast growth factor receptor 3
FGFR4	Fibroblast growth factor receptor 4
GAREM1	GRB2 associated regulator of MAPK1 subtype 1
GAS6	Growth arrest specific 6
GATA3	GATA binding protein 3
GO	Gene Ontology

GPNMB	Glycoprotein Nmb
GPR183	G protein-coupled receptor 183
GSEA	Gene Set Enrichment Analysis
GWAS	Genome Wide Association Study
HAND2	Heart and neural crest derivatives expressed 2
HLA-B	Major histocompatibility complex, class I, B
HLA-DPA1	Major histocompatibility complex, class II, DP alpha 1
HLA-DQB1	Major histocompatibility complex, class II, DQ beta 1
HLA-DR(B1/A/B5)	Major histocompatibility complex, class II, DQ beta 1/ alpha/ beta 5
HMGCR	3-hydroxy-3-methylglutaryl-CoA reductase
IFN-γ	Interferon gamma
IFNGR2	Interferon gamma receptor 2
INPP5D	Inositol polyphosphate-5-phosphatase D
IRF(4/5/8)	Interferon regulatory factor 4/5/8
JAK2	Janus kinase 2
KCNN4	Potassium calcium-activated channel subfamily N member 4
LINGO1	Leucine rich repeat and Ig domain containing 1
LRRTM3	Leucine rich repeat transmembrane neuronal 3
NAT2	N-acetyltransferase 2
NECAB2	N-terminal EF-hand calcium binding protein 2
NECTIN2	Nectin cell adhesion molecule 2
NELFE	Negative elongation factor complex member E
NFKB1	Nuclear factor kappa B subunit 1
NOD2	Nucleotide binding oligomerization domain containing 2
NOTCH2	Notch receptor 2
OASL	2'-5'-oligoadenylate synthetase like
OPRM1	Opioid receptor mu 1
PAK2	p21 (RAC1) activated kinase 2
PDE4D	Phosphodiesterase 4D
PDGFC	Platelet derived growth factor C
PHLDA3	Pleckstrin homology like domain family A member 3
PLC(G1/G2)	Phospholipase C gamma 1/ 2
PRKD2	Protein kinase D2
PTK2B	Protein tyrosine kinase 2 beta
PTPN11/22	Protein tyrosine phosphatase non-receptor type 11/22
RAB29	RAB29, member RAS oncogene family
RAF1	Raf-1 proto-oncogene, serine/threonine kinase
RAP1A	RAP1A, member of RAS oncogene family
RASGRP1	RAS guanyl releasing protein 1
SCIMP	SLP adaptor and CSK interacting membrane protein
SKAP1	src kinase associated phosphoprotein 1
TAB2	TGF-beta activated kinase 1 (MAP3K7) binding protein 2
TGFB1	Transforming growth factor beta 1
TNFRSF11A	TNF receptor superfamily member 11a
TP53 or p53	Tumor protein p53
TP63	Tumor protein p63
TREM2	Triggering receptor expressed on myeloid cells 2
TRIM(5/8/26/31)	Tripartite motif containing 5/8/26/31
VEGFA	Vascular endothelial growth factor A

References

1 Vijg, J. & Suh, Y. Genome instability and aging. *Annu Rev Physiol* **75**, 645-668, doi:10.1146/annurev-physiol-030212-183715 (2013).

2 Herrmann, M., Pusceddu, I., Marz, W. & Herrmann, W. Telomere biology and age-related diseases. *Clin Chem Lab Med* **56**, 1210-1222, doi:10.1515/cclm-2017-0870 (2018).

3 Ormazabal, V. *et al.* Association between insulin resistance and the development of cardiovascular disease. *Cardiovasc Diabetol* **17**, 122, doi:10.1186/s12933-018-0762-4 (2018).

4 Krahenbuhl, S. Mitochondria: important target for drug toxicity? *J Hepatol* **34**, 334-336, doi:10.1016/s0168-8278(00)00106-9 (2001).

5 Deeks, S. G. HIV infection, inflammation, immunosenescence, and aging. *Annu Rev Med* **62**, 141-155, doi:10.1146/annurev-med-042909-093756 (2011).

6 Aiello, A. *et al.* Immunosenescence and Its Hallmarks: How to Oppose Aging Strategically? A Review of Potential Options for Therapeutic Intervention. *Front Immunol* **10**, 2247, doi:10.3389/fimmu.2019.02247 (2019).

7 Murata, T., Ohtsuka, C. & Terayama, Y. Increased mitochondrial oxidative damage and oxidative DNA damage contributes to the neurodegenerative process in sporadic amyotrophic lateral sclerosis. *Free Radic Res* **42**, 221-225, doi:10.1080/10715760701877262 (2008).

8 Lobetti-Bodoni, C. *et al.* Telomere loss in Philadelphia-negative hematopoiesis after successful treatment of chronic myeloid leukemia: evidence for premature aging of the myeloid compartment. *Mech Ageing Dev* **133**, 479-488, doi:10.1016/j.mad.2012.05.007 (2012).

9 Luebeck, E. G. *et al.* Identification of a key role of widespread epigenetic drift in Barrett's esophagus and esophageal adenocarcinoma. *Clin Epigenetics* **9**, 113, doi:10.1186/s13148-017-0409-4 (2017).

10 Mohanty, B. P., Bhattacharjee, S., Paria, P., Mahanty, A. & Sharma, A. P. Lipid biomarkers of lens aging. *Appl Biochem Biotechnol* **169**, 192-200, doi:10.1007/s12010-012-9963-6 (2013).

11 Dagres, N. *et al.* Insulin sensitivity and coronary vasoreactivity: insulin sensitivity relates to adenosine-stimulated coronary flow response in human subjects. *Clin Endocrinol (Oxf)* **61**, 724-731, doi:10.1111/j.1365-2265.2004.02156.x (2004).

12 Zhang, H., Liu, Y., Lao, M., Ma, Z. & Yi, X. Puerarin protects Alzheimer's disease neuronal cybrids from oxidant-stress induced apoptosis by inhibiting pro-death signaling pathways. *Exp Gerontol* **46**, 30-37, doi:10.1016/j.exger.2010.09.013 (2011).

13 Liu, R., Liu, H., Ha, Y., Tilton, R. G. & Zhang, W. Oxidative stress induces endothelial cell senescence via downregulation of Sirt6. *Biomed Res Int* **2014**, 902842, doi:10.1155/2014/902842 (2014).

14 Chang, H. X., Yang, L., Li, Z., Chen, G. & Dai, G. Age-related biological characterization of mesenchymal progenitor cells in human articular cartilage. *Orthopedics* **34**, e382-388, doi:10.3928/01477447-20110627-06 (2011).

15 Li, D. *et al.* Kinetics of tumor necrosis factor alpha in plasma and the cardioprotective effect of a monoclonal antibody to tumor necrosis factor alpha in acute myocardial infarction. *Am Heart J* **137**, 1145-1152, doi:10.1016/s0002-8703(99)70375-3 (1999).

16 Partridge, L. & Gems, D. Mechanisms of ageing: public or private? *Nat Rev Genet* **3**, 165-175, doi:10.1038/nrg753 (2002).
